# Atmospheric water vapor contribution to interannual variability of Northern Hemisphere summer heatwaves

**DOI:** 10.1038/s41612-026-01361-4

**Published:** 2026-02-28

**Authors:** Dingrui Cao, Hai Lin, Yi Huang

**Affiliations:** 1 Department of Atmospheric and Oceanic Sciences, McGill University, Montreal, QC Canada; 2 Recherche en Prévision Numérique Atmosphérique, Environment and Climate Change Canada, Montreal, QC Canada

**Keywords:** Climate sciences, Environmental sciences

## Abstract

Previous studies suggested that atmospheric water vapor, as a greenhouse gas, can amplify surface warming by enhancing downwelling longwave radiation (DLR), thereby potentially intensifying extreme heatwaves. However, the atmospheric water vapor anomalies associated with heatwaves and their radiative effects remain poorly quantified. Here, using radiative kernels to decompose DLR anomalies, we demonstrate that atmospheric water vapor exerts a substantial influence on summertime interannual-scale heatwave-related DLR across the Northern Hemisphere, despite DLR being largely dominated by near-surface air warming. This influence exhibits pronounced regional contrasts: mid‑ to high‑latitude heatwaves are typically accompanied by moist air, which enhances DLR, whereas heatwaves in India and western North America often coincide with drier conditions that reduce DLR and thereby partially offset the positive DLR anomalies caused by air warming. The dryness over India is linked to an interannual weakening of the Indian summer monsoon, while that over western North America is associated with soil‑moisture deficits. Our findings provide a quantitative assessment of the contribution of atmospheric moisture to extreme heatwaves.

## Introduction

Climate change has intensified summer extreme heatwaves (EHWs) worldwide, producing unprecedented events across the Northern Hemisphere (NH), such as those in Europe in 2019^1^, the western U.S. in 2021^2,3^, and the Yangtze River Basin in China in 2022^4,5^. These heatwaves have generated cascading societal impacts, including elevated heat-related mortality, severe agricultural losses, and strains on energy systems. Addressing this growing threat, therefore, requires a comprehensive understanding of heatwave characteristics and their underlying mechanisms to inform risk mitigation, climate-resilient planning, and evidence-based policy.

Atmospheric water vapor is the most potent natural greenhouse gas: as its concentration increases, downwelling longwave radiation (DLR) is enhanced, thereby contributing to surface warming^6,7^. By the Clausius–Clapeyron relation, a warmer atmosphere can hold more moisture; nevertheless, during EHWs the spatial distribution of atmospheric moisture across the NH is often complex and highly heterogeneous^8^. This complexity largely reflects the interplay between diverse atmospheric circulation anomalies, varying regional evaporation and land-surface conditions^9–11^.

Several recent studies have shown that variations in dynamical and land-surface processes modulate moisture, thereby shaping heatwave characteristics across subregions. For example, the North Pacific–North America wave train can amplify EHWs in the western United States by inducing subsidence that reduces cloud cover and increases downwelling shortwave radiation; concurrent moisture transport from the subtropical Pacific elevates atmospheric water vapor and strengthens surface DLR, compounding the warming^12,13^. Another example is the persistence of anticyclonic anomalies over northern East Asia since the late 1990s, which has intensified soil moisture deficits, limited evaporative cooling, and consequently amplified surface warming while reinforcing the blocking pattern^14^.

Compound heatwave events exhibit substantial diversity in their moisture environments, and previous studies have shown that their moisture signatures are closely tied to distinct atmospheric dynamics and surface energy‑flux responses^14–16^. In East Asia, EHWs occurring under reduced atmospheric moisture are often linked to amplified anticyclonic circulation associated with midlatitude wave trains, which enhances adiabatic warming and increases incoming shortwave radiation through cloud suppression. By contrast, moisture-abundant conditions during EHWs can arise from locally intensified anticyclonic anomalies, where subsidence elevates atmospheric moisture and DLR, promoting moisture‑related cloud feedback that further reinforces surface warming^17^. Similar contrasts have been documented in North America: moisture-enhanced events are frequently associated with moisture transport from the North Pacific and elevated DLR, whereas moisture-deficient events tend to be dominated by increased shortwave radiation associated with reduced total cloud cover over land^13^. This land-based reduction in cloudiness in continental heatwave environments differs from the persistent marine low-cloud decks typically found in subtropical eastern ocean basins under large-scale subsidence^18^. More broadly, moisture‑limited heatwaves are common in semi‑arid to semi‑humid regions, particularly in the subtropics, where persistent high pressure suppresses clouds, strengthens solar heating, deepens soil‑moisture deficits, and shifts surface energy partitioning toward sensible heat. In contrast, moisture‑abundant environments such as the tropics and high latitudes more often experience heatwaves accompanied by elevated atmospheric moisture, as ample soil moisture supports evaporative cooling that partially offsets solar‑driven warming under similar high‑pressure conditions^8^.

Previous work has also shown that large-scale circulation anomalies can substantially modulate the redistribution of atmospheric water vapor^19,20^, and that regional soil moisture–atmosphere feedbacks strongly influence atmospheric moisture content and surface fluxes^21–23^. Through land–atmosphere coupling, these moisture anomalies induce changes in surface radiation and thereby affect land surface temperature and EHW occurrence^6,12,24,25^. Despite these advances, two key gaps remain. First, the physical mechanisms that drive regional differences in moisture responses during NH EHWs are not yet fully understood. Second, quantitative assessments of how atmospheric moisture anomalies modify the surface energy budget during NH EHWs are scarce. This study focuses on the interannual variability of summertime heatwave frequency and its association with seasonal‑mean anomalies in atmospheric conditions, with heatwaves identified using a 90th‑percentile daily maximum temperature threshold, and addresses two key questions: (1) how large-scale dynamical forcing and regional land–atmosphere interactions together shape the seasonal-mean atmospheric moisture anomalies associated with more frequent NH EHWs; and (2) how strongly these seasonal-mean atmospheric water vapor anomalies influence surface radiative fluxes during summers with higher EHW frequency.

## Results

### Concurrent atmospheric water vapor anomalies and heatwave types across the Northern Hemisphere

**Fig. 1:**
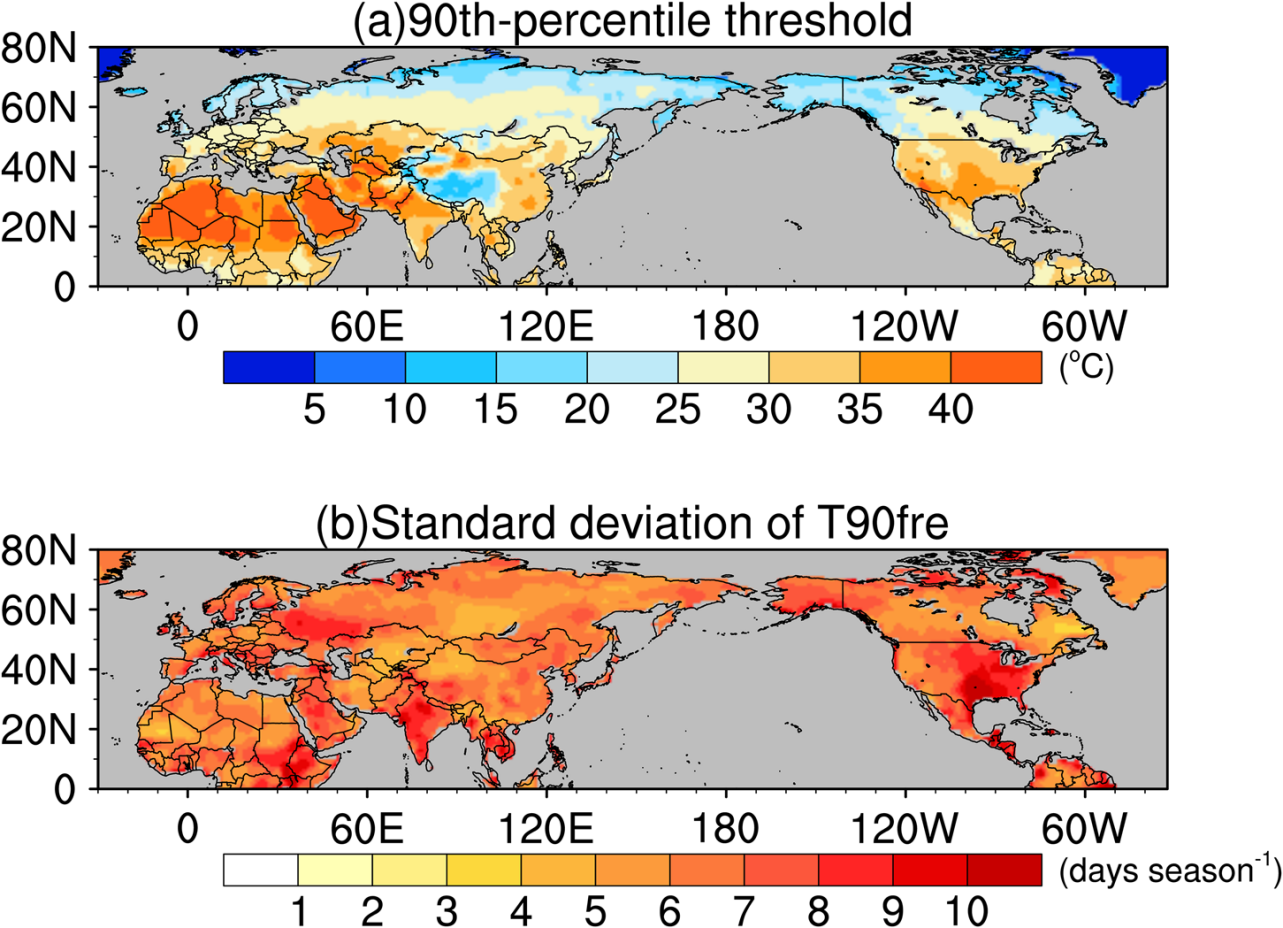
Boreal summer heatwave metrics during 1981–2020. **a** 90th‑percentile threshold of daily maximum 2-m temperature (shading; °C) and **b** interannual standard deviation of seasonal heatwave frequency (shading; days season^−1^).

Results from ERA5 reanalysis indicate that, across the NH, EHW thresholds generally decrease with increasing latitude (Fig. 1a). However, the Tibetan Plateau exhibits significantly lower temperatures than other regions at similar latitudes due to its high elevation. In contrast, desert regions such as northern Africa and the Arabian Peninsula interior show much higher EHW thresholds, largely due to the influence of the subtropical high (Supplementary Fig. 1a), which maintains cloud-free conditions (Supplementary Fig. 1b) that allow strong shortwave solar radiation to reach the surface. To quantify interannual variability in EHW occurrence, we use T90fre, defined as the June–July–August (JJA) count of days with daily maximum 2‑m temperature exceeding the 90th‑percentile threshold. The standard deviation of T90fre reveals prominent centers of variability over northern Europe, India, Southeast Asia, and the southeastern United States, indicating large interannual fluctuations in EHW frequency across these regions (Fig. 1b).

**Fig. 2:**
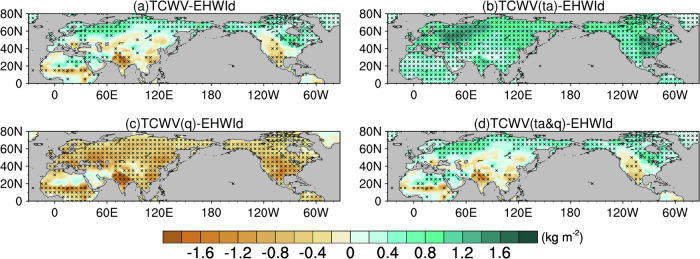
Total column water vapor (TCWV) anomalies associated with extreme heatwaves. Pointwise regressions onto the co‑located EHWI_d_ for **a** TCWV, **b** temperature‑related TCWV, **c** moisture‑supply‑related TCWV, and **d** total anomalies obtained by summing the temperature‑ and moisture‑supply‑related components. Shading denotes TCWV anomalies in kg m^−2^, and black crosses mark grid points significant at the 95% confidence level (Student’s *t* test).

Here, we examine total column water vapor (TCWV) anomalies associated with EHW frequency and use these anomalies to classify heatwave types across NH subregions. We quantify interannual variability in EHW frequency using a detrended EHW index (EHWI_d_; see “Methods”), and then perform pointwise regressions of TCWV anomalies onto the co-located EHWI_d_ at each grid cell (Fig. 2a). We classify heatwaves into three types according to the sign and statistical significance of their co‑located TCWV anomalies: humid heatwaves, characterized by significantly positive TCWV anomalies; dry heatwaves, characterized by significantly negative anomalies; and neutral heatwaves, for which TCWV anomalies are not statistically distinguishable from zero. Overall, atmospheric water vapor anomalies over NH land areas exhibit a pronounced latitudinal contrast. EHWs in the mid‑ to high latitudes typically fall into the humid‑heatwave category, whereas those in India and western North America (WNA) are representative of dry heatwaves. However, this latitudinal tendency does not apply uniformly across all subregions. For instance, EHWs in northern Africa and the interior of the Arabian Peninsula are associated with increased TCWV and therefore classify as humid heatwaves, while in the southeast United States (SEUS), TCWV anomalies are minimal, leading to their designation as neutral heatwaves.

Distinct heatwave types across the NH indicate that local atmospheric moisture does not always scale with temperature as predicted by the Clausius–Clapeyron relation. In addition to the thermodynamic effect of warming, which increases the saturation vapor capacity, atmospheric circulation and land–atmosphere interactions can substantially modify moisture conditions during EHWs. To separate these influences, we decompose TCWV anomalies (Fig. 2a, d) into two components. The temperature‑related TCWV reflects the component of TCWV associated with changes in saturation moisture content under local warming (${\mathrm{\varDelta TCWV}}_{T}$, see Fig. 2b), representing the temperature‑dependent portion of atmospheric moisture. The moisture‑supply‑related TCWV describes the atmospheric moisture surplus or deficit relative to the saturation level at a fixed temperature (${\mathrm{\varDelta TCWV}}_{q}$, see Fig. 2c), corresponding to the relative humidity (RH)‑dependent portion of atmospheric moisture. Details of the decomposition procedure are provided in Supplementary Note 1.

We next summarize NH-wide interannual patterns revealed by decomposing EHW-related TCWV anomalies into a temperature-related component (Fig. 2b) and a moisture-supply-related component (Fig. 2c). The temperature-related contribution is consistently positive across the NH, reflecting increased saturation moisture capacity under anomalous warming, although its magnitude varies markedly among subregions. By contrast, the moisture-supply-related contribution is negative over most land areas, indicating a moisture deficit relative to saturation when temperature effects are held fixed. The net TCWV anomaly during EHWs is therefore governed by the balance between these two terms and the degree to which they offset one another. Humid heatwaves occur when temperature-driven moistening exceeds the opposing moisture-supply-related drying, yielding increased TCWV. Dry heatwaves arise when moisture-supply-related drying dominates, producing decreased TCWV. Neutral heatwaves emerge when the two contributions are comparable and largely canceled, leaving TCWV anomalies near zero. This decomposition thus provides a unified, physically interpretable framework for linking distinct heatwave types to different balances between thermodynamic moistening and moisture-supply limitations.

Across individual subregions, the relative importance of the two components varies systematically. In India and WNA, two representative dry‑heatwave regions, the moisture‑supply‑related drying strongly exceeds the temperature‑related moistening, leading to net decreases in atmospheric moisture during EHWs. By contrast, across mid‑ to high‑latitude regions of the NH, the temperature‑related contribution dominates: its positive effect on TCWV substantially outweighs the opposing supply‑related drying, producing increased atmospheric moisture during EHWs. In northern Africa and the interior Arabian Peninsula, this dominance is even more pronounced, with observed TCWV increases arising almost entirely from the temperature‑related component and only a negligible contribution from moisture supply. In SEUS, by contrast, temperature‑related moistening and moisture‑supply‑related drying are of comparable magnitude and largely cancel each other, producing minimal net change in atmospheric moisture during EHWs.

This thermodynamic supply framework aligns with previous work that separates water vapor radiative effects into fixed‑RH and varying‑RH components. For example, a previous study decomposed clear‑sky greenhouse‑effect anomalies into contributions from temperature‑driven changes in saturation capacity and circulation‑driven variations in RH, demonstrating that the spatial pattern of water vapor radiative anomalies is dominated by the RH‑varying term^26^. Their El Niño-Southern Oscillation composites further show that circulation‑induced moisture anomalies can reverse the sign of the total water vapor contribution between El Niño and La Niña. Viewed alongside our results, these findings reinforce the interpretation that humid and dry heatwaves arise from different combinations of thermodynamic moistening and circulation or land‑surface constraints, which ultimately shape the sign and magnitude of the longwave radiative response during EHWs.

**Fig. 3:**
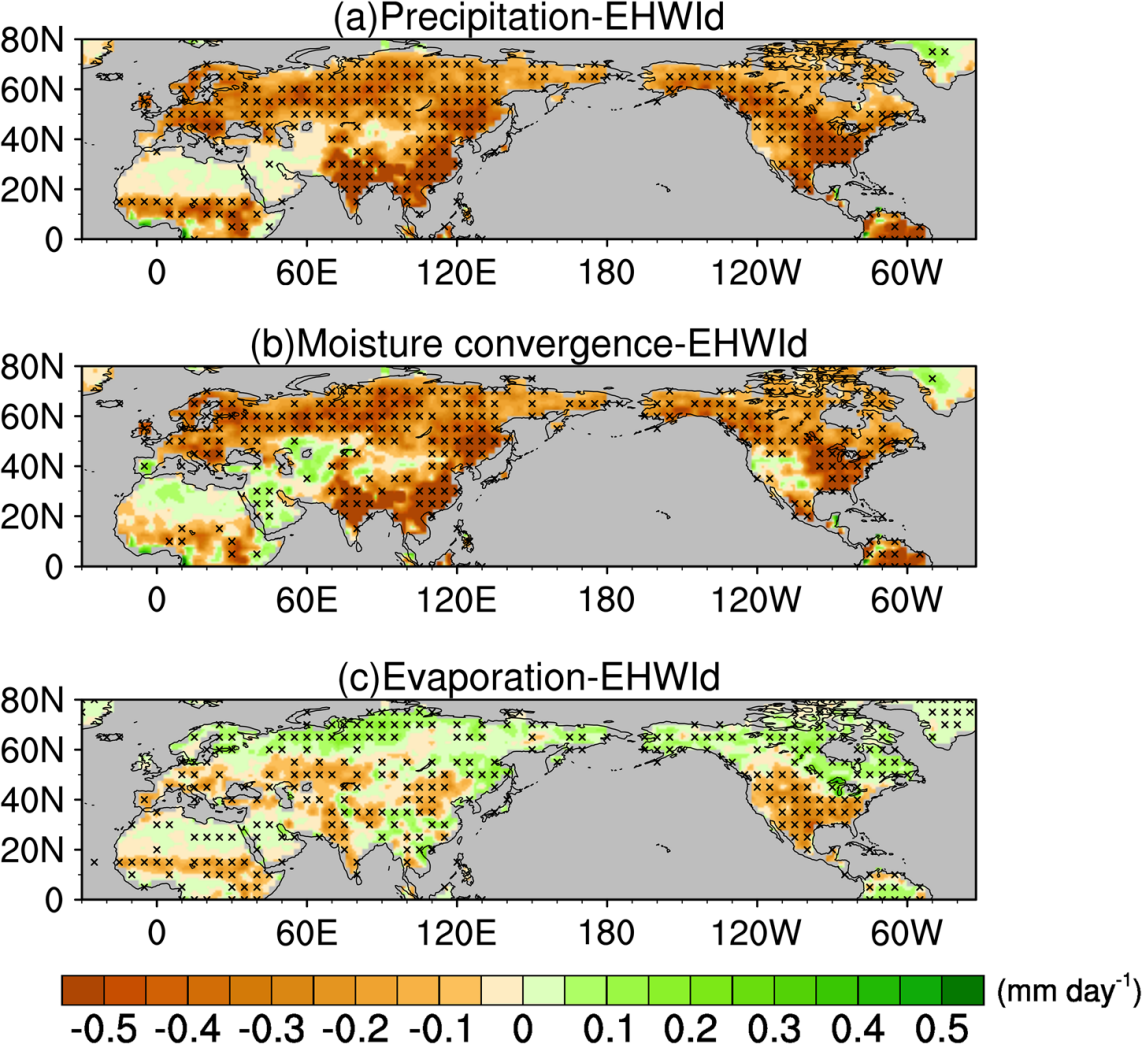
Moisture budget anomalies associated with extreme heatwaves. Pointwise regressions onto the co‑located EHWI_d_ for **a** precipitation, **b** moisture convergence ($-\nabla \cdot \vec{Q}$), and **c** surface evaporation. Shading denotes values in mm day^−1^ for each panel, and black crosses mark grid points significant at the 95% confidence level (Student’s *t* test).

Using the TCWV decomposition framework described above, we quantified the contributions of temperature-related and moisture-supply-related processes to atmospheric moisture anomalies during NH EHWs. The temperature-related component represents changes in atmospheric saturation vapor pressure associated with temperature perturbations. In contrast, the moisture‑supply component isolates the portion of the specific humidity anomaly arising from changes in RH, quantified relative to the climatological saturation field. This term, therefore, captures moisture surpluses or deficits associated with RH departures. Consequently, the spatial pattern of the moisture-supply-related component often resembles precipitation anomalies during EHWs (Fig. 3a). We next decompose the moisture-supply-related component further, via the moisture budget analysis, into circulation-induced moisture transport (Fig. 3b) and local evaporation (Fig. 3c) to elucidate mechanisms that give rise to diverse heatwave types across the NH. Across most NH subregions, enhanced moisture divergence is the dominant contributor to negative anomalies in the moisture‑supply component, although northern Africa, the interior Arabian Peninsula, WNA, and West Asia form notable exceptions. Relative to moisture divergence across subregions during EHWs, the role of regional surface evaporation is generally much smaller. In NH mid- to high-latitudes, reductions in atmospheric moisture driven by enhanced moisture divergence far outweigh compensating increases from local evaporation. Although evaporation typically plays a secondary role within the moisture-supply-related component, its contribution is non-negligible in certain regions. For example, in WNA, weak moisture divergence implies that the negative moisture-supply-related anomalies there are primarily attributable to reduced surface evaporation. In SEUS, by contrast, enhanced divergence and diminished surface evaporation act together to exacerbate the local moisture deficit.

### Regional mechanisms underlying heatwave‑related moisture anomalies

To examine how moisture-supply-dominated dry heatwaves arise in contrasting regional settings, we focus on two illustrative cases: India and WNA. Although both regions experience dry heatwaves, the processes driving their moisture deficits differ markedly, reflecting their distinct climatological backgrounds.

**Fig. 4:**
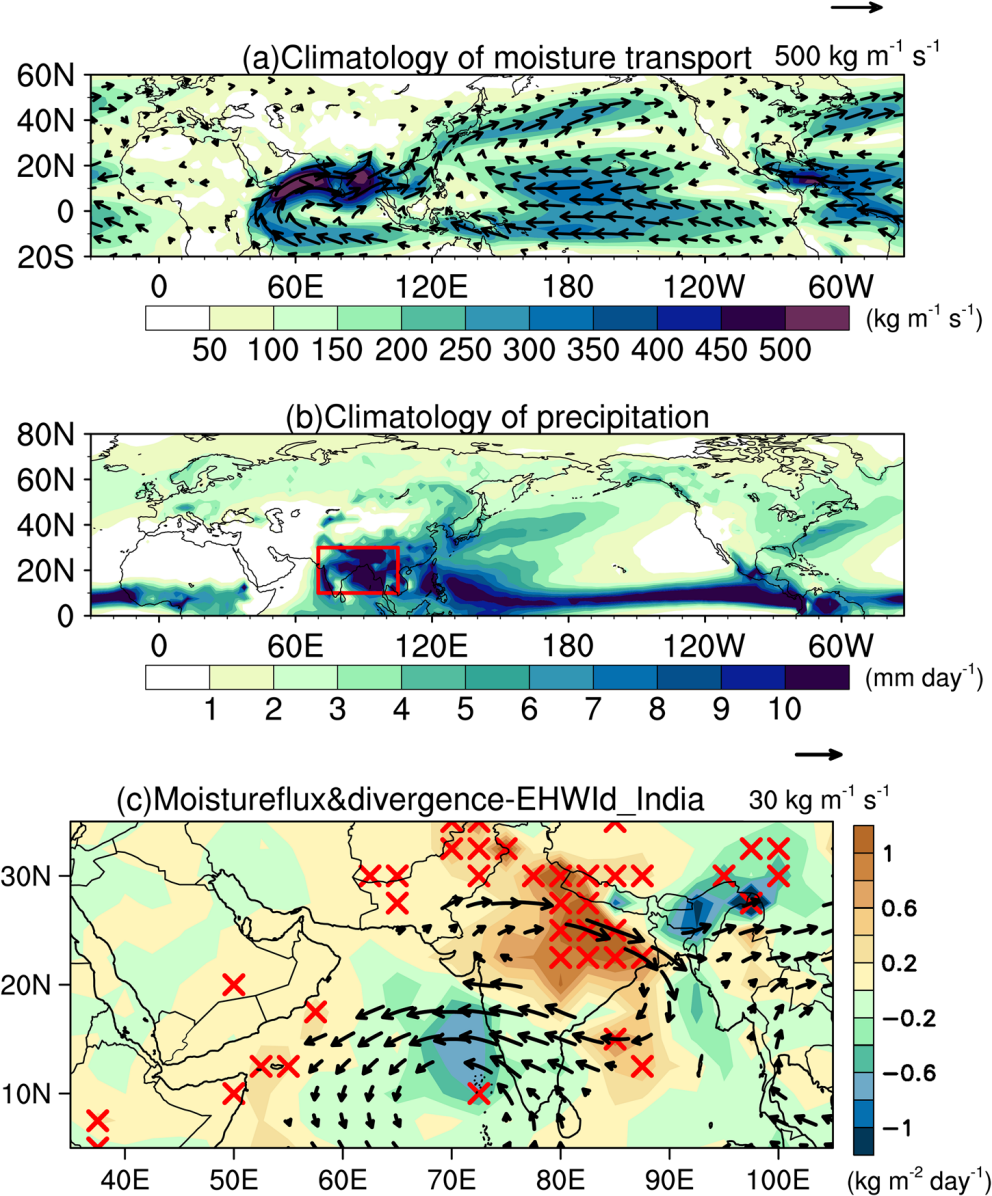
Climatological moisture flux, precipitation, and moisture flux divergence associated with extreme heatwaves in India. Mean-state JJA fields: **a** vertically integrated moisture flux (vectors; kg m^−1^ s^−1^) and flux magnitude (shading; kg m^−1^ s^−1^), and **b** precipitation (shading; mm day^−1^). **c** As in **a** but showing anomalies of vertically integrated moisture flux (vectors; kg m^−1^ s^−1^) and flux divergence (shading; kg m^−2^ day^−1^) regressed onto the Indian EHWI_d_. The red rectangle in **b** highlights the key Indian summer monsoon region (70°E − 105°E, 10°N − 30°N). Red crosses in **c** denote values significant at the 95% confidence level (Student’s *t* test). Only vectors with magnitudes exceeding **a** 50 kg m^−1^ s^−1^ and **c** 5 kg m^−1^ s^−1^ are plotted.

In India, summer conditions are dominated by the Indian summer monsoon (ISM). Climatologically, a strong land-sea temperature contrast between the Indian subcontinent and the Indian Ocean drives vigorous southwesterly flow in the lower troposphere, delivering abundant moisture and precipitation to India (Fig. 4a, b). Previous studies showed that ISM onset can mitigate heatwave intensity over India^27–29^. To explore this relationship on the interannual timescale, we define the ISM Index (ISMI) as the standardized mean precipitation over 70°E − 105°E and 10°N − 30°N during JJA^30^ (red box in Fig. 4b). The correlation coefficient between ISMI and the Indian EHWI_d_ (68°E–90°E, 15°N–30°N) is −0.44, statistically significant at the 0.05 level according to a Student’s *t* test, indicating that stronger heatwaves coincide with a weakened monsoon. Moisture transport anomalies during Indian EHWs reveal a cyclonic circulation over the Indian Ocean that suppresses southwesterly flow and impedes northward moisture transport to India, consistent with a weakened monsoon. Meanwhile, an anticyclonic anomaly north of India enhances moisture divergence, reinforcing dry-heatwave conditions (Fig. 4c). In our analysis, the monsoon weakening associated with EHWs reflects interannual anomalies within the study period. By contrast, long-term ISM changes arise from different external forcings: historical weakening has been linked to anthropogenic aerosols, whereas greenhouse gas-driven warming is generally expected to increase monsoon rainfall^31^. These forced responses involve mechanisms that are distinct from the interannual ISM–heatwave linkage examined here.

**Fig. 5:**
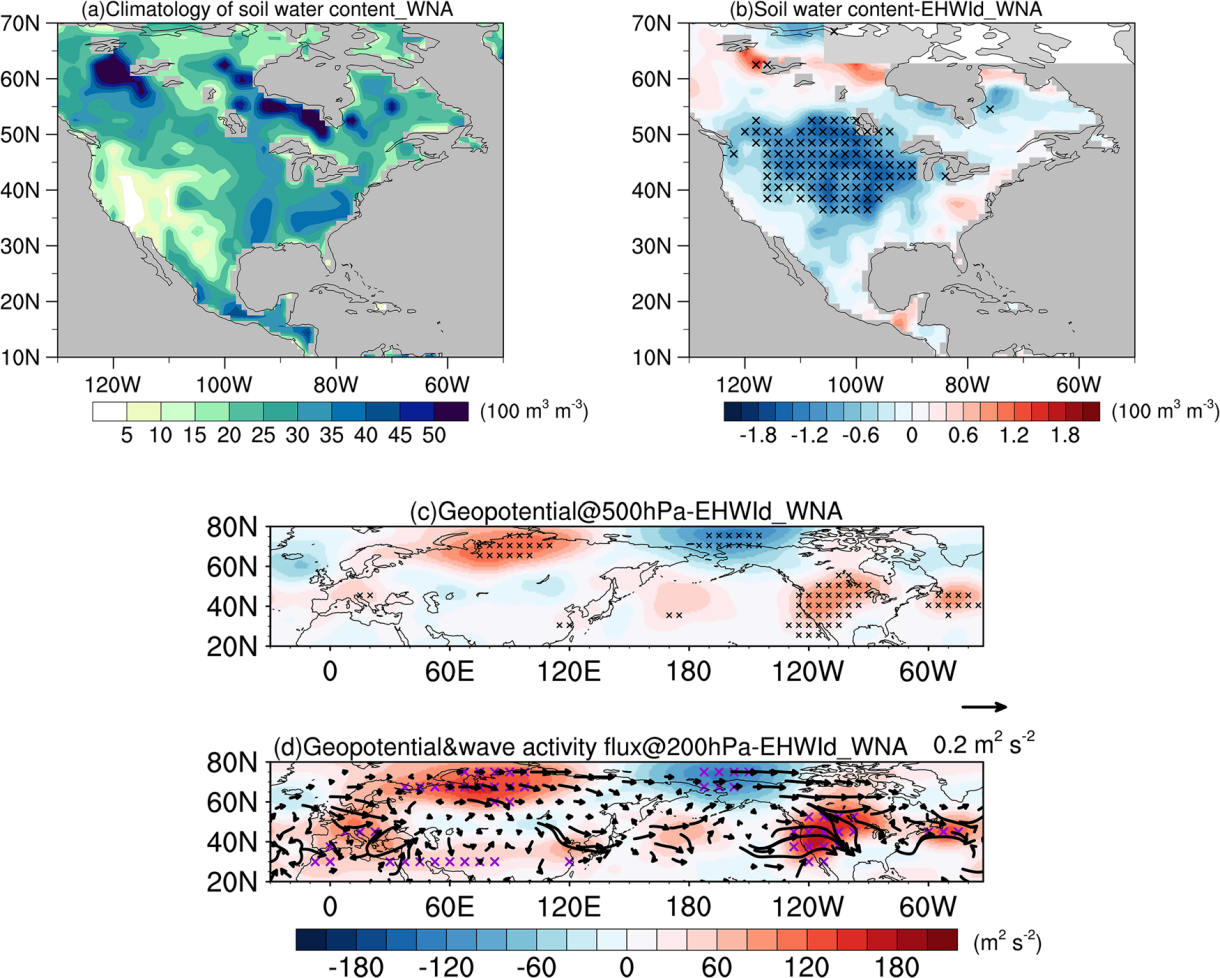
Soil water content and geopotential associated with extreme heatwaves. **a** Climatological JJA soil water content (shading; 100 m^3^ m^−3^), **b** soil water content anomalies, pointwise regressed onto the EHWI_d_ (shading; 100 m^3^ m^−3^), **c** 500-hPa geopotential anomalies regressed onto the western North America (WNA) EHWI_d_ (shading; m^2^ s^−2^) and **d** 200-hPa geopotential anomalies (shading; m^2^ s^−2^) and wave activity flux (vectors; m^2^ s^−2^) regressed onto the WNA EHWI_d_. In **b**, **d**, black and red crosses indicate values that are statistically significant at the 95% confidence level (Student’s *t* test), respectively.

Compared with India’s humid climate, WNA is an arid region with low background soil water content (Fig. 5a), resulting in a moisture-limited evaporation regime during EHWs. Regressions of 500-hPa and 200-hPa geopotential anomalies onto the WNA EHWI_d_ (100°W–125°W, 20°N–50°N) reveal two quasi-barotropic wave trains originating from high-latitude Eurasia (Fig. 5c, d). The stronger wave train is centered over Siberia as a positive geopotential anomaly, extends eastward with a negative anomaly over Alaska, and ultimately penetrates into WNA as a positive anomaly. A weaker wave train also originates over Siberia and propagates southeastward through Mongolia and the northwestern Pacific before reaching WNA, where it reinforces the positive geopotential anomaly. The positive geopotential anomalies over WNA correspond to an amplified high-pressure ridge that can directly warm the surface. Positive geopotential anomalies over Siberia are likely linked to the interannual variability of Arctic sea ice^32^. These two wave trains thus provide sources of interannual variability for heatwaves in WNA. Heatwave-induced soil moisture depletion (Fig. 5b) constrains surface evaporation rates in WNA (Fig. 3c). The consequent reduction in atmospheric water vapor reinforces the dry-heatwave pattern observed during EHWs.

In NH mid‑ to high‑latitude regions, such as northern Europe, Siberia, and northeastern Canada, abundant background soil water content (Supplementary Fig. 1c) places evaporation in an energy‑limited regime, enabling surface warming during EHWs to substantially enhance evaporation. In addition, the relatively low temperatures in these regions increase the Clausius–Clapeyron sensitivity, so that a given amount of warming produces a larger fractional increase in saturation vapor pressure. Combined with the high background humidity, which facilitates the realization of this thermodynamic moistening, atmospheric moisture rises markedly during EHWs. Although NH mid‑ to high‑latitude heatwaves are accompanied by subsidence (Supplementary Fig. 2a) and moisture divergence (Fig. 3b), our decomposition shows that these dynamic drying effects are weaker than the thermodynamic moistening from surface warming and enhanced evaporation, resulting in a net increase in atmospheric moisture.

By contrast, although northern Africa and the interior Arabian Peninsula experience humid heatwaves, moisture‑supply processes are remarkably weak in these regions. Both moisture divergence and surface evaporation show only minimal anomalies, indicating that moisture transport and surface evaporation play little role in the moistening observed during EHWs. Instead, the increase in atmospheric water vapor primarily reflects a thermodynamic response, whereby surface warming raises the saturation vapor content of the atmosphere.

In SEUS, reduced evaporation combined with enhanced moisture divergence acts to lower atmospheric water vapor during EHWs (Fig. 3b, c). However, this drying is largely offset by temperature-driven moistening (Fig. 2b), resulting in little net change in regional atmospheric moisture. Unlike WNA, SEUS has relatively high background soil moisture (Fig. 5a), so a warming-induced increase in evaporation is physically plausible. At the same time, strong subsidence over SEUS (Supplementary Fig. 2a) can suppress evaporation (Fig. 3c) and induce local moisture divergence (Fig. 3b). Notably, JRA-55 and ERA5 disagree on the sign of evaporation anomalies in SEUS: JRA-55 indicates increased evaporation while ERA5 does not (Supplementary Fig. 12c). This discrepancy suggests that subsidence-induced suppression (Supplementary Figs. 2a and 12e) and warming-induced enhancement of evaporation may compete in SEUS, leaving evaporation anomalies during extreme heatwaves uncertain across reanalysis products.

### Quantifying atmospheric water vapor contributions to surface radiative fluxes associated with heatwave frequency

Atmospheric water vapor, as a potent greenhouse gas, can enhance DLR and thereby promote surface warming. However, increased atmospheric water vapor can also attenuate incoming solar radiation. In the following analysis, we investigate the effects of water vapor on DLR and net surface shortwave radiation (SWR), respectively, during NH EHWs, to determine whether its net effect is warming or cooling.

Here, we perform pointwise regressions of anomalies in DLR and SWR onto the co-located EHWI_d_ at each grid point. Notably, SWR shows significant positive anomalies over most of the NH landmass, excluding northern Africa, the Arabian Peninsula interior, and the Mongolia Plateau (Fig. 7a). This significant increase in surface solar radiation during EHWs is closely linked to a concurrent reduction in total cloud cover (TCC) (Supplementary Fig. 2b).

**Fig. 6:**
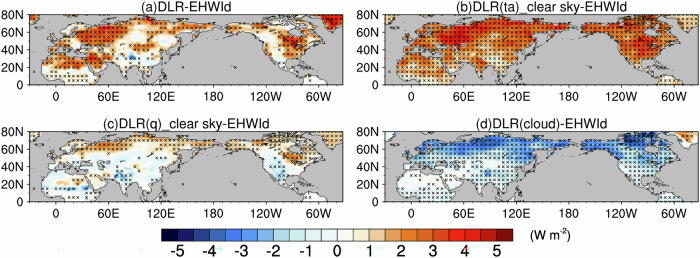
Anomalies of surface downwelling longwave radiation (DLR) associated with extreme heatwaves. Pointwise regressions onto the co‑located EHWI_d_ for **a** DLR, and DLR attributed to **b** air temperature, **c** atmospheric water vapor, and **d** clouds determined as the difference between all‑sky and clear‑sky DLR. Shading denotes DLR anomalies in W m^−2^, and black crosses mark grid points significant at the 95% confidence level (Student’s *t* test).

Compared with SWR, anomalies in DLR are smaller in magnitude but display pronounced contrasts across different heatwave types (Fig. 6a). Typical humid heatwaves, occurring over the NH mid‑ to high‑latitudes as well as northern Africa and the interior Arabian Peninsula, are characterized by notable increases in DLR, consistent with the strong greenhouse effect of atmospheric water vapor in these regions. In contrast, dry‑heatwave regions such as India and WNA exhibit only minor DLR changes. Substantial DLR increases are also observed over the Mongolia Plateau and SEUS, despite minimal anomalies in atmospheric water vapor; these areas represent neutral‑heatwave regimes. Previous studies have shown that DLR is influenced not only by atmospheric moisture but also by factors such as cloud cover and air temperature^33^. The combined effects of these variables contribute to the distinct spatial patterns of DLR anomalies across humid, dry, and neutral heatwaves.

Here, to quantify DLR responses to changes in air temperature, water vapor and clouds, we apply ERA5-based radiative kernels^34^. First, using clear-sky kernels, we isolate the individual contributions of air temperature and atmospheric water vapor to DLR. Next, we compute the cloud contribution as the difference between all-sky and clear-sky DLR. Finally, summing up these three contributions produces a total DLR pattern that closely matches the observed all-sky anomalies. This correspondence confirms that the ERA5 kernels accurately quantify the impacts of these atmospheric variables on DLR.

Our results show that air temperature and TCC are the dominant contributors to DLR during EHWs. Increased air temperature produces positive DLR anomalies across the entire NH (Fig. 6b), while reductions in TCC (Supplementary Fig. 2b) generate negative DLR anomalies (Fig. 6d). Although the influence of atmospheric water vapor on DLR is smaller in magnitude, it remains non-negligible (Fig. 6c). Different heatwave types exhibit distinct patterns of DLR anomalies. In typical humid‑heatwave regions, including the NH mid‑ to high‑latitudes and northern Africa and the interior Arabian Peninsula, enhanced TCWV contributes positively to DLR. By contrast, in dry‑heatwave regions such as India and WNA, decreases in atmospheric water vapor exert a negative influence on DLR, counteracting the warming‑induced increase in DLR. As a result, despite substantial surface warming, total DLR shows little net change over India and WNA (Supplementary Fig. 5a). In neutral‑heatwave regions, such as the Mongolian Plateau and the SEUS, where water vapor anomalies are minimal, the increase in DLR is driven almost entirely by air temperature changes. Building on these kernel‑derived DLR components, we translate the DLR anomalies into their corresponding contributions to skin temperature (SKT) anomalies using the Stefan–Boltzmann relationship. This step directly links the radiative perturbations to surface warming or cooling. It also shows that the spatial patterns of the water‑vapor‑ and air‑temperature‑related SKT anomalies closely follow those of their respective DLR anomalies (Supplementary Fig. 3). This framework allows us to quantify how much of the surface warming arises from water vapor‑driven versus air temperature‑driven longwave radiative changes.

We further examined how water vapor modifies the DLR response relative to the combined effects of air temperature and clouds. The spatial pattern of total DLR changes markedly when the water vapor contribution is removed (Supplementary Fig. 5): the southwest–to–northeast contrast over North America disappears, yielding a more uniform DLR increase across western and central regions with little change in eastern Canada. A similar effect occurs over India, where DLR increases once the water vapor contribution is omitted. These comparisons demonstrate that water vapor anomalies play a central role in shaping the regional structure of DLR during EHWs.

Notably, during EHWs, the spatial patterns of DLR responses attributable to air temperature derived from all-sky kernels (Supplementary Figs. 6a and 7a) closely resemble those from clear-sky kernels (Fig. 6b), although all-sky responses are slightly larger in magnitude, especially across mid- to high latitudes. By contrast, the spatial patterns of DLR responses attributable to water vapor are likewise highly consistent, but all-sky responses are marginally weaker (Supplementary Figs. 6b and 7b), again most clearly in mid- to high latitudes. In the kernel framework, these differences should be interpreted as the influence of the climatological cloud field embedded in the all‑sky kernels, rather than as a representation of radiative responses under anomalous cloud conditions (e.g., departures in effective cloud cover from the climatological mean). Accordingly, the all‑sky results are used here solely in a mechanistic sense to illustrate how mean‑state clouds modulate the underlying radiative sensitivities relative to clear‑sky conditions. Within this mechanistic interpretation, the contrasting temperature and water vapor responses arise from two distinct cloud radiative effects^33^. First, the increase in temperature-kernel sensitivity under all-sky conditions occurs because clouds, acting as highly efficient longwave blackbody emitters, amplify the downward radiative signal of temperature perturbations originating within or adjacent to the cloud layer (Supplementary Fig. 9e). Second, in the lower troposphere, the sensitivity of all-sky water-vapor kernels is reduced relative to clear-sky kernels owing to cloud masking: clouds absorb and re-emit longwave radiation and thereby obscure the radiative contribution of humidity perturbations aloft (Supplementary Fig. 9f).

We further quantified the contributions of boundary-layer air temperature (Supplementary Fig. 8a) and boundary-layer water vapor (Supplementary Fig. 8b) to DLR and compared them with the corresponding contributions from the entire tropospheric column (Fig. 6b, c). The spatial patterns and magnitudes of the boundary-layer air temperature contributions closely match those of the full troposphere, indicating that the air temperature effect on DLR is largely confined to the boundary layer. This finding is consistent with the vertical structure of the air temperature radiative kernels for DLR, which are dominated by atmospheric layers at pressures below approximately 850 hPa throughout the NH (Supplementary Fig. 9a). By contrast, although the spatial patterns of the boundary layer and full-column water vapor contributions are similar, the magnitudes of the boundary-layer contributions are substantially smaller, especially poleward of about 50°N, implying that water vapor above the boundary layer makes a nontrivial contribution to DLR. This interpretation is supported by the vertical profile of the water vapor radiative kernels for DLR, which show appreciable sensitivity above the near-surface layer, between about 850 and 700 hPa, in NH mid and high latitudes (Supplementary Fig. 9b).

**Fig. 7:**
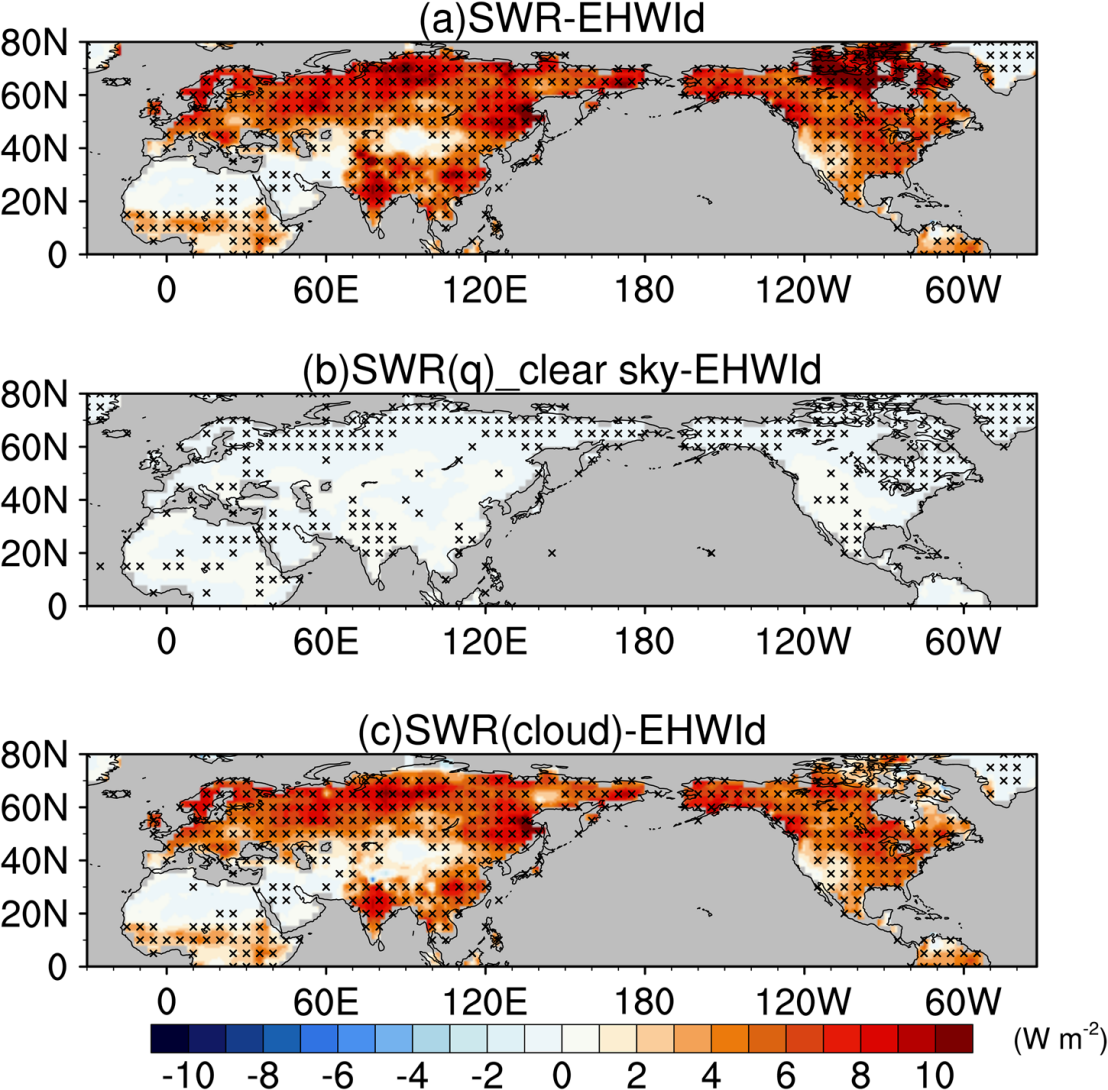
Anomalies of net surface shortwave radiation (SWR) associated with extreme heatwaves. Pointwise regressions onto the co‑located EHWI_d_ for **a** SWR, and SWR attributed to **b** atmospheric water vapor and **c** clouds determined as the difference between all‑sky and clear‑sky SWR. Shading denotes SWR anomalies in W m^−2^, and black crosses mark grid points significant at the 95% confidence level (Student’s *t* test).

We next quantify the contribution of atmospheric water vapor to SWR during NH EHWs. Using clear‑sky radiative kernels, we isolate the water vapor‑related component of SWR and diagnose the cloud contribution as the difference between all‑sky and clear‑sky SWR. The results show that decreases in TCC during EHWs allow more solar radiation to reach the surface (Supplementary Fig. 2b), making cloud changes the dominant driver of the SWR increase (Fig. 7c), whereas the water vapor contribution is negligible and does not influence the spatial pattern of SWR anomalies (Fig. 7b). We then translate the SWR anomalies into their corresponding contributions to SKT anomalies. The resulting SKT patterns closely mirror the SWR patterns, with cloud‑driven SWR increases producing the primary SWR‑related surface warming and water vapor‑related SWR‑induced SKT changes remaining minimal (Supplementary Fig. 4).

## Discussion

Focusing on the interannual variability of seasonal‑mean atmospheric anomalies associated with seasonal‑scale EHW frequency, this study identifies distinct interannual‑scale moisture conditions linked to different types of NH heatwaves. We classify mid- to high-latitude regions as humid-heatwave areas, where frequent heatwaves are accompanied by moist air primarily driven by enhanced surface evaporation and surface warming; in these regions, the higher moisture further increases DLR. By contrast, some mid- to low-latitude regions, most notably India and WNA, exhibit moisture declines during EHWs and are categorized as dry-heatwave regions. In India, the moisture reduction is linked to a weakened ISM, whereas in WNA it primarily reflects persistently low background soil moisture. Notably, despite pronounced moisture decreases in these dry-heatwave regions, DLR remains largely unchanged. In addition, SEUS falls into the neutral‑heatwave category, where reduced evaporation and enhanced moisture divergence are largely offset by temperature‑driven moistening, leading to little net change in atmospheric water vapor during EHWs.

To quantify the relative contributions of air temperature, atmospheric water vapor, and clouds to DLR during EHWs, we applied ERA5-based radiative kernels. Our results indicate that surface warming uniformly enhances DLR across the NH, while reductions in TCC act to suppress it. Although the magnitude of the water vapor contribution is smaller than that of temperature, it remains significant. In humid‑heatwave regions, increases in atmospheric water vapor together with rising air temperature jointly enhance DLR during EHWs. In contrast, in dry‑heatwave regions such as India and WNA, reductions in atmospheric water vapor offset the warming‑driven increase in DLR, resulting in little net DLR change. In neutral-heatwave regions, including the Mongolian Plateau and the SEUS, atmospheric moisture exhibits little net change; consequently, increases in air temperature emerge as the dominant contributor to DLR increases. In addition, we also quantify the contribution of atmospheric water vapor to SWR during EHWs. Compared with its substantial influence on DLR, the water vapor contribution to SWR is minimal, while the increase in SWR is primarily driven by cloud reductions. Because water vapor plays only a negligible role in modulating SWR, the SWR response shows little variation across the different humidity‑defined heatwave types, and SWR anomalies are therefore not a major focus of this study.

A notable feature revealed in this study is the contrasting moisture anomalies that emerge along the mid‑latitude westerlies during NH EHWs: atmospheric water vapor generally increases across mid‑ to high‑latitude land areas but decreases in lower‑latitude regions such as India and WNA. This meridional contrast may reflect differences in dominant dynamical timescales. NH mid‑ to high‑latitude regions are strongly shaped by synoptic‑scale transient eddies^35^, while tropical moisture sources exert little influence at these latitudes^36^. The rapid moist adjustment associated with these disturbances prevents substantial accumulation of anomalous moisture transport. Consequently, moisture anomalies in these regions primarily reflect a temperature‑driven moistening component. In contrast, lower‑latitude regions are more strongly modulated by low‑frequency variability. For example, weakened monsoonal inflow over India reduces moisture transport into the region, favoring moisture declines during EHWs. These mechanisms remain hypotheses and require targeted process‑level analysis, including separating high‑ and low‑frequency dynamical contributions and quantitatively diagnosing moisture transport and adjustment pathways during EHWs.

This study emphasizes local radiative and moisture responses within individual subregions and does not explicitly quantify interregional coupling. Future work could examine how large-scale circulation patterns, such as the circumglobal teleconnection^37–39^, modulate heatwave variability across regions and thereby influence associated moisture–radiation interactions across the NH.

## Methods

### Data and analysis

This study utilizes June-July-August (JJA) atmospheric variables from the fifth generation of atmospheric reanalysis (ERA5) of the European Centre for Medium-Range Weather Forecasts (ECMWF) spanning 1981−2020^40^. Daily maximum 2-m air temperature data are used to diagnose EHWs, while monthly variables, including vertically integrated water vapor, total cloud cover (TCC), precipitation, evaporation, air temperature, geopotential, boundary layer height, 2-m dewpoint temperature, skin temperature, wind velocities, surface pressure, volumetric soil moisture (layer 1), horizontal water vapor fluxes, and surface heat fluxes, are analyzed to quantify their anomalies associated with EHWs. These variables are analyzed on 2.5° × 2.5° grids^12^. This study examines the relationship between atmospheric circulation, surface heat fluxes, and EHWs on interannual timescales, with long-term trends removed to isolate year-to-year variability.

To quantify the contributions of atmospheric water vapor, air temperature, and clouds to surface radiative fluxes, we employ ERA5-based radiative sensitivity kernels^34^. These kernels provide vertically resolved sensitivities of (1) DLR to unit perturbations in water vapor and air temperature, and (2) SWR to unit perturbations in water vapor only, under both clear-sky and all-sky conditions. The clear-sky kernels isolate direct radiative effects, while the all-sky kernels additionally capture cloud–radiation interactions. The radiative kernels are provided at a 2.5° × 2.5° horizontal resolution across 37 pressure levels (1−1000 hPa).

To ensure the robustness of ERA5-derived findings, we conduct cross-validation by comparing atmospheric variable anomalies during EHWs between the Japanese 55-year Reanalysis (JRA-55)^41^ and ERA5 reanalysis datasets. Details of the results derived from the JRA‑55 reanalysis are provided in the Supplementary Figs. 12 and 13, while the main text highlights only those aspects where the JRA‑55 results differ from ERA5.

### Definition of extreme heatwave events

EHWs are defined as days when the daily maximum 2‑m temperature (Tmax) exceeds the climatological 90th‑percentile threshold for that calendar day (TX90). To obtain TX90, we smooth the seasonal cycle of the threshold by pooling Tmax samples from the target day and its ±2 neighboring days across all years, forming a centered 5‑day window^12^. We then compute the seasonal EHW frequency (T90fre), defined as the number of days in each JJA that exceed the TX90 threshold. To quantify interannual variability in T90fre, we first compute anomalies relative to the 1981–2020 climatological mean and then standardize these anomalies using their interannual standard deviation, yielding the EHW index (EHWI). We then remove the long-term trend from EHWI via linear regression, obtaining the detrended index (EHWI_d_), which filters out warming signals and highlights year-to-year atmospheric fluctuations. To quantify the interannual co-variability between heatwave frequency and the summer-mean atmospheric and surface conditions, we regress JJA seasonal-mean anomalies of atmospheric circulation and surface energy flux variables onto the detrended heatwave-frequency index (EHWI_d_). The resulting regression patterns represent the typical summer-mean anomalies in years with anomalous heatwave frequency. Seasonal-mean anomalies are computed from monthly fields to characterize the interannual variability of the JJA seasonal-mean anomalies, consistent with the seasonal definition of EHWI_d_ and the year-to-year focus of this analysis.

### Atmospheric moisture variability attributable to temperature and moisture supply

To quantify the contributions of temperature and non-temperature driven processes to atmospheric moisture anomalies during EHWs, we decompose specific humidity anomalies ($dq$) into temperature- ($T$) and relative humidity- ($\mathrm{RH}$) related components:

1 $${dq}={{dq}}_{T}+{{dq}}_{\mathrm{RH}}$$

2 $${{dq}}_{T}=\frac{{L}_{v}{q}_{0}{dT}}{{R}_{v}{T}_{0}^{2}}$$

3 $$d{q}_{\mathrm{RH}}={q}_{\mathrm{sat}}d\mathrm{RH}$$

where ${q}_{0}$ and ${T}_{0}$ are $q$ and $T$ in climatology, respectively, ${q}_{\mathrm{sat}}$ is the saturation specific humidity.

Although $\mathrm{RH}$ depends on $T$, $d{q}_{\mathrm{RH}}$ can be regarded as the moisture change caused by non-temperature-related processes. Its variations primarily reflect atmospheric moisture deficit or surplus (see Supplementary Note 1 for details).

To quantify changes in total column water vapor ($\mathrm{\varDelta TCWV}$) attributable to $T$ and $\mathrm{RH}$ variations, we vertically integrate the components $d{q}_{T}$ and $d{q}_{\mathrm{RH}}$ from the surface pressure (${Ps}$) to the upper-tropospheric pressure (${Pt}$ = 1 hPa) as follows:

4 $$\Delta TCWV={\Delta TCWV}_{T}+{\Delta TCWV}_{q}$$

5 $${\mathrm{\varDelta TCWV}}_{T}=\frac{1}{g}{\int }_{{Pt}}^{{Ps}}{{dq}}_{T}{dP}$$

6 $$\Delta TCW{V}_{q}=\frac{1}{g}{\int }_{{Pt}}^{{Ps}}{{dq}}_{\mathrm{RH}}{dP}$$

where ${\Delta TCWV}_{T}$ denotes the portion of $\mathrm{TCWV}$ change due only to $T$ variations, representing the temperature-related saturation capacity contribution. $\Delta {{\mathrm{TCWV}}}_{q}$ denotes the moisture-supply portion, the change in $\mathrm{TCWV}$ resulting from the local moisture budget with temperature held constant. A detailed derivation of Eqs. (1)–(6) is provided in Supplementary Note 1.

### Moisture budget

Moisture budget analysis is used to diagnose atmospheric water vapor changes attributable to moisture-supply-related processes, such as moisture transport and evaporation^42,43^. On interannual timescales, the moisture budget can be expressed as follows:

7 $$P-E=-\nabla \cdot \vec{Q,}$$

where $P$ and $E$ are precipitation rate and evaporation rate from the surface, respectively, $\nabla \cdot \vec{Q}$ is the moisture flux divergence.

### Land surface energy budget

We analyze surface heat flux anomalies during EHWs using the land surface energy budget^8^, expressed as:

8 $$ULR=SWR+DLR-LH-SH-Q,$$

where $\mathrm{ULR}$ is the surface upward longwave radiation, $\mathrm{SWR}$ is the net surface shortwave radiation, and $\mathrm{DLR}$ is the downwelling longwave radiation. All radiative fluxes in the right-hand side of Eq. (8) are defined as positive when directed downward. Surface turbulent heat fluxes are represented by $\mathrm{LH}$ and $\mathrm{SH}$, both defined as positive when directed upward. The ground heat flux $Q$ is included for completeness, but is negligible compared to the other terms and is therefore not discussed further.

In this study, we mainly focus on $\mathrm{SWR}$ and $\mathrm{DLR}$, as well as the contributions of atmospheric water vapor to them.

### Surface radiative fluxes calculated via radiative kernels

To quantify the radiative contributions of air temperature and water vapor to DLR, and of water vapor to SWR, we employ the kernel-based methodology^33^ utilizing radiative sensitivity kernels^31^. Specifically, the contributions of air temperature and specific humidity to DLR under clear-sky and all-sky conditions, as well as the contributions of specific humidity to SWR under the same conditions, are calculated by vertically integrating the product of the radiative kernels and the corresponding atmospheric perturbation profiles, from the surface to the top of the atmosphere (1 hPa). These contributions are represented as follows:

9 $${\Delta DLR}_{{T}}={\int }_{{Pt}}^{{Ps}}\frac{{K}_{k}^{T}d{T}_{k}}{100hPa}{dP}$$

10 $${\Delta DLR}_{q}={\int }_{{Pt}}^{{Ps}}\frac{{K}_{k}^{q({lw})}d{T}_{k}^{q}}{100hPa}{dP}$$

11 $${\Delta SWR}_{q}={\int }_{{Pt}}^{{Ps}}\frac{{K}_{k}^{q({sw})}d{T}_{k}^{q}}{100hPa}{dP}$$

Here, ${K}_{k}^{T}$ and ${K}_{k}^{q({lw})}$ are the air temperature and specific humidity radiative kernels for DLR at pressure level $k$, and ${K}_{k}^{q({sw})}$ is the specific humidity radiative kernel for SWR at level $k$. All kernels are given per unit perturbation and are reported with units of W m^−2^ K^−1^ (100 hPa)^−1^. ${Ps}$ and ${Pt}$ denote the surface pressure and the upper-level pressure, both in hPa; $d{T}_{k}$ is the air temperature anomaly at level $k$, and $d{T}_{k}^{q}$ denotes the temperature-equivalent representation of a specific humidity perturbation at the level *k*, as used in the standard radiative-kernel framework^34^.

Based on the vertical sensitivity structure of DLR kernels^33^, radiative sensitivities are concentrated in the lower troposphere, with air temperature kernels dominating below 850 hPa (Supplementary Fig. 9a) and water vapor kernels dominating below 700 hPa (Supplementary Fig. 9b). To investigate whether DLR anomalies during EHWs primarily originate in the near-surface layer, we redefine the upper integration limit ${Pt}$ in Eqs. (9) and (10) as the boundary layer top pressure (${{Pt}}_{{BL}}$), defined as:

12 $${\Delta DLR}_{T}={\int }_{{{Pt}}_{{BL}}}^{{Ps}}\frac{{K}_{k}^{T}d{T}_{k}}{100hPa}{dP}$$

13 $${\Delta DLR}_{q}={\int }_{{{Pt}}_{{BL}}}^{{Ps}}\frac{{K}_{k}^{q({lw})}d{T}_{k}^{q}}{100hPa}{dP}$$

The procedure for converting the ERA5 boundary-layer height (${Z}_{{BL}}$) to the corresponding ${{Pt}}_{{BL}}$ is described in Supplementary Note 2.

### Quantifying the contributions of water vapor and air temperature to surface warming

To relate the diagnosed radiative anomalies directly to surface warming, we quantify their contributions to skin temperature (SKT) by linking surface flux perturbations to temperature changes through the Stefan–Boltzmann law^8^. Starting from the surface energy budget (Eq. (8)), anomalies in ULR are given by:

14 $$\Delta URL=\Delta SWR+\Delta DLR-\Delta LH-\Delta SH-\Delta Q$$

The upward longwave emission satisfies $\mathrm{ULR}=\sigma {\mathrm{SKT}}^{4}$, where $\sigma$ is the Stefan–Boltzmann constant. Linearizing around the climatological mean $\overline{\mathrm{SKT}}$ yields a first-order conversion from $\Delta ULR$ to $\Delta SKT$:

15 $$\Delta SKT=\frac{\Delta ULR}{4\sigma {\overline{SKT}}^{3,}}$$

Combining Eqs. (14) and (15) provides an additive attribution of $\mathrm{\varDelta SKT}$ to individual surface flux components. In particular, the contributions associated with DLR and SWR are expressed as:

16 $${\mathrm{\varDelta SKT}}_{\mathrm{DLR}}=\frac{\mathrm{\varDelta DLR}}{4\sigma \overline{{\mathrm{SKT}}^{3}}}$$

17 $${\mathrm{\varDelta SKT}}_{\mathrm{SWR}}=\frac{\mathrm{\varDelta SWR}}{4\sigma \overline{{\mathrm{SKT}}^{3}}}$$

Because the prefactor $\frac{1}{4\sigma \overline{{\mathrm{SKT}}^{3}}}$ is common to all terms, the kernel-based decomposition of $\mathrm{\varDelta DLR}$ and $\mathrm{\varDelta SWR}$ into components driven by atmospheric water vapor and air temperature can be directly translated into additive surface warming components, e.g., ${\mathrm{\varDelta SKT}}_{\mathrm{DLR}q}=\frac{{\mathrm{\varDelta DLR}}_{q}}{4\sigma \overline{{\mathrm{SKT}}^{3}}}$ and ${\mathrm{\varDelta SKT}}_{\mathrm{DLR}{ta}}=\frac{{\mathrm{\varDelta DLR}}_{{ta}}}{4\sigma \overline{{\mathrm{SKT}}^{3}}}$, and analogously for SWR. Furthermore, ${\mathrm{\varDelta SKT}}_{\mathrm{DLR}}$ is partitioned into air temperature, humidity, and cloud components plus a residual term (${\mathrm{\varDelta SKT}}_{\mathrm{DLR}{res}}$):

18 $$\Delta SK{T}_{{D}{L}{R}}={\Delta SKT}_{DLRta}+{\Delta SKT}_{DLRq}+{\Delta SKT}_{DLRcld}+{\Delta SKT}_{DLRres}$$

The cloud-modulated component is diagnosed as the difference between all-sky and clear-sky DLR (SWR). The residual term is reported for completeness but is not discussed further because it is small relative to the other components.

### Wave activity flux

The wave activity flux is widely used to diagnose the sources and propagation of stationary Rossby waves^44^. The horizontal flux is defined by the following equation:

19 $$W=\frac{P}{2\left|\vec{U}\right|}\left\{\begin{array}{c}U\left({v}^{{\prime} 2}-{\psi }^{{\prime} }{v}_{x}^{{\prime} }\right)+V(-{u}^{{\prime} }{v}^{{\prime} }+{\psi }^{{\prime} }{u}_{x}^{{\prime} })\\ U\left(-{u}^{{\prime} }{v}^{{\prime} }+{\psi }^{{\prime} }{u}_{x}^{{\prime} }\right)+V\left({u}^{{\prime} 2}-{\psi }^{{\prime} }{v}_{y}^{{\prime} }\right)\end{array}\right.$$

where $P$ denotes the pressure scaled by 1000 hPa, $\vec{U}=(U,V)$ represents the basic‑state wind velocity, $\vec{{V}^{{\prime} }}=({u}^{{\prime} },{v}^{{\prime} })$ refers to the perturbed wind velocity, and ${\psi }^{{\prime} }$ is the perturbation geostrophic streamfunction.

## Supplementary information

Supplementary Information

## Data Availability

ERA5 reanalysis data were obtained from the Copernicus Climate Data Store (https://cds.climate.copernicus.eu/datasets). JRA-55 reanalysis data were accessed via the NCAR Research Data Archive (https://rda.ucar.edu/datasets). ERA5 radiative kernels are available at 10.17632/vmg3s67568.4.

## Code availability

The code used in this study is available from the corresponding author upon reasonable request.
