# Supplementary material for: Atmospheric water vapor contribution to interannual variability of Northern Hemisphere summer heatwaves
Source: NPJ Clim Atmos Sci. 2026 Feb 28;9(1):88. doi: 10.1038/s41612-026-01361-4 (PMC13065471; doi:10.1038/s41612-026-01361-4)
Supplement: Supplementary file 1 — Supplementary Information [file 41612_2026_1361_MOESM1_ESM.pdf]

Supplementary Information

Atmospheric Water Vapor Contribution to Interannual Variability of Northern Hemisphere Summer Heatwaves

Dingrui Cao [1*], Hai Lin [1, 2] and Yi Huang [1]

[1] Department of Atmospheric and Oceanic Sciences, McGill University, Montreal, Quebec, Canada

[2] Recherche en Prévision Numérique Atmosphérique, Environment and Climate Change Canada, Montreal, Quebec, Canada

*Corresponding author: Dingrui Cao, Department of Atmospheric and Oceanic Sciences, McGill University, Montreal, Quebec, Canada, Email: dingrui.cao@mcgill.ca

Supplementary Note 1: Atmospheric moisture variability attributable to temperature and moisture supply

We decompose the total change in total column water vapor (TCWV) into two conditional components: (1) the temperature-related saturation-capacity component, denoted $\Delta TCWV_T$, defined as the change in TCWV resulting solely from air temperature variations with the relative humidity held constant. $\Delta TCWV_T$ is computed from the Clausius–Clapeyron relation to quantify the temperature dependence of saturation specific humidity; and (2) the moisture supply-related component, denoted $\Delta TCWV_q$, defined as the change in TCWV resulting from the local moisture budget with air temperature held constant, which primarily reflects the local moisture deficit or surplus. Below we list the assumptions and outline the derivation.

The mixing ratio of a moist air sample (r) is defined as the mass of water vapor (m_v) divided by the mass of dry air (m_d) in that sample, expressed as:

$$r = \frac{m_v}{m_d}, \quad (1)$$

The relationship between r and vapor pressure (e) can be expressed as:

$$r = \frac{0.622e}{P-e}, \quad (2)$$

where P is the air pressure.

The relationship between q and r can be expressed as:

$$q = \frac{m_v}{m_v+m_d} = \frac{r}{r+1}, \quad (3)$$

Since the $r \ll 1$, it can be approximate as:

$$q \approx r = \frac{0.622e}{P-e}, \quad (4)$$

The relationship between saturation vapor pressure (e_s) and e can be expressed as:

$$e = e_s RH, \quad (5)$$

where RH is relative humidity.

According to the empirical relation, the dependence of e_s on air temperature (T) can be expressed as:

$$e_s(T) = 6.112\, exp(\frac{17.67\,(T-273.15)}{(T-273.15)+243.15}), \quad (6)$$

According to Eqs. (4) and (5), q can be expressed as:

$$q = \frac{0.622 RH e_s(T)}{P - RH e_s(T)}, \quad (7)$$

According to Eq. (7), q is essentially a function of T and RH, and can be written concisely as:

$$q = q(T, RH), \quad (8)$$

Variations in q can be expressed as:

$$dq = \frac{\partial q}{\partial T} dT + \frac{\partial q}{\partial RH} dRH, \quad (9)$$

$$dq = dq_T + dq_{RH}, \quad (10)$$

where dq_T denotes the component of q anomalies arising from changes in T with RH held at its climatological value, and dq_{RH} denotes the component arising from changes in RH with T held at its climatological value.

Here, dq_T is related to the Clausius–Clapeyron relation, which quantifies the exponential dependence of $e_s(T)$ on T. Specifically, it can be expressed as:

$$\frac{de_s(T)}{e_s(T)} = \frac{L_v dT}{R_v T^2}, \quad (11)$$

where L_v is the latent heat of vaporization ($L_v = 2.5 \times 10^6$ J kg^{-1}), R_v is the gas constant for water vapor ($R_v = 461.5$ J kg^{-1} K^{-1})

According to Eq. (4), saturation specific humidity (q_{sat}) can be expressed as:

$$q_{sat} = \frac{0.622 e_s(T)}{P - e_s(T)}, \quad (12)$$

Since $P \gg e_s(T)$ and $P \gg RH e_s(T)$, Eqs. (7) and (12) simplify the q_{sat} and q to:

$$q = \frac{0.622 RH e_s(T)}{P}, \quad (13)$$

$$q_{sat} = \frac{0.622 e_s(T)}{P}, \quad (14)$$

Therefore, since q_{sat}, q and $e_s(T)$ are proportional to each other, the changes in q due to air temperature changes can be approximated as:

$$dq_T = \frac{L_v q_0 dT}{R_v T_0^2}, \quad (15)$$

where q_0 and T_0 are q and T in climatology, respectively.

Taking the partial derivative of Eq. (7) with respect to RH, the RH-driven component (dq_{RH}) is given by

$$dq_{RH} = \frac{0.622Pe_s(T)}{[P-RHe_s(T)]^2}dRH, \quad (16)$$

Since $P \gg RHe_s(T)$, Eq. (16) simplifies the dq_{RH} to

$$dq_{RH} = \frac{0.622e_s(T)}{P}dRH, \quad (17)$$

According to Eq. (14), dq_{RH} can be expressed as:

$$dq_{RH} = q_{sat}dRH, \quad (18)$$

According to Eq. (5), RH is fundamentally a function of e and e_s and can be expressed succinctly as:

$$RH = RH(e_s, e), \quad (19)$$

Based on Eqs. (13) and (14), q is directly proportional to e, while q_{sat} is directly proportional to e_s. Accordingly, Eq. (19) can be expressed as:

$$RH = \frac{q}{q_{sat}} = RH(q_{sat}, q), \quad (20)$$

Variations in RH can be expressed as:

$$dRH = \frac{\partial RH}{\partial q}dq + \frac{\partial RH}{\partial q_{sat}}dq_{sat}, \quad (21)$$

$$dRH = dRH_q + dRH_{q_{sat}}, \quad (22)$$

$$dRH = \frac{dq}{q_{sat}} - \frac{q}{q_{sat}^2}dq_{sat}, \quad (23)$$

where $dRH_q = \frac{dq}{q_{sat}}$ denotes the component of RH anomalies arising from changes in q with q_{sat} held at its climatological value, and $dRH_{q_{sat}} = \frac{q}{q_{sat}^2}dq_{sat}$ denotes the component arising from changes in q_{sat} (or T) with q held at its climatological value.

According to Eq. (6), variations in e_s can be expressed as:

$$de_s = \frac{de_s}{dT}dT, \quad (24)$$

Given that q_{sat} is directly proportional to e_s, Eq. (24) can be expressed as:

$$dq_{sat} = \frac{dq_{sat}}{dT} dT, \quad (25)$$

Eq. (23) can be reformulated as:

$$dRH = \frac{dq}{q_{sat}} - \frac{q}{q_{sat}^2} \frac{dq_{sat}}{dT} dT, \quad (26)$$

In light of Eq. (11), Eq. (26) can be reformulated as:

$$dRH = \frac{dq}{q_{sat}} - \frac{q}{q_{sat}} \frac{L_v}{R_v T^2} dT, \quad (27)$$

$$q_{sat} dRH = dq - \frac{L_v q dT}{R_v T^2} = dq - dq_T = dq_{RH}, \quad (28)$$

Although RH depends on T, according to Eqs. (18) and (28), dq_{RH} can be regarded as the moisture change caused by non-temperature-related processes. Its variations primarily reflect atmospheric moisture deficit or surplus.

To quantify changes in $TCWV$ attributable to T and RH variations, we vertically integrate the components dq_T and dq_{RH} from the surface pressure (Ps) to the upper-tropospheric pressure (Pt = 1 hPa) as follows:

$$\Delta TCWV = \Delta TCWV_T + \Delta TCWV_q, \quad (29)$$

$$\Delta TCWV_T = \frac{1}{g} \int_{Pt}^{Ps} dq_T dP, \quad (30)$$

$$\Delta TCWV_q = \frac{1}{g} \int_{Pt}^{Ps} dq_{RH} dP, \quad (31)$$

where $\Delta TCWV_T$ denotes the portion of $TCWV$ change due only to T variations, representing the temperature-related saturation capacity contribution. $\Delta TCWV_q$ denotes the moisture-supply portion, the change in $TCWV$ resulting from the local moisture budget with temperature held constant.

Supplementary Note 2: Surface radiative fluxes calculated via radiative kernels

To investigate whether DLR anomalies during EHWs primarily originate in the near-surface layer, we redefine the upper integration limit Pt as the boundary layer top pressure (Pt_{BL}), defined as:

$$\Delta DLR_T = \int_{Pt_{BL}}^{Ps} \frac{K_k^T dT_k}{100hPa} dP, \quad (32)$$

$$\Delta DLR_q = \int_{Pt_{BL}}^{Ps} \frac{K_k^{q(lw)} dT_k^q}{100hPa} dP, \quad (33)$$

ERA5 provides the boundary layer height (Z_{BL}) but not the corresponding pressure. We therefore estimate the Pt_{BL} from the hypsometric equation:

$$Pt_{BL} = Ps\ exp\left(-\frac{g\ Z_{BL}}{R_d \overline{T_v}}\right), \quad (34)$$

where R_d is the specific gas constant for dry air (R_d = 287.05J kg^{-1} K^{-1}), g is the acceleration of gravity (g = 9.8m s^{-2}), $\overline{T_v}$ is the log-pressure-weighted mean virtual temperature of the column between the surface pressure (Ps) and the boundary layer top pressure (Pt_{BL}), defined as:

$$T_{vk} = (T_k + 0.61 q_k), \quad (35)$$

$$\overline{T_v} = \frac{\int_{Pt_{BL}}^{Ps} T_{vk} dP}{\int_{Pt_{BL}}^{Ps} dP}, \quad (36)$$

where T_{vk} is the virtual temperature at pressure level k and q_k is the specific humidity at level k.

However, because Pt_{BL} is the unknown to be determined, identifying the appropriate upper pressure bound for computing $\overline{T_v}$ is challenging. As a practical approximation for a well-mixed convective boundary layer, the $\overline{T_v}$ can be estimated from surface parameters. We therefore approximate:

$$\overline{T_v} \approx T_v = (T_{2m} + 0.61 q_s), \quad (37)$$

where T_{2m} and q_s are 2-m temperature and surface specific humidity, respectively.

The vertical profile of the zonally averaged T_v is shown in Supplementary Figure 10. The results indicate that, within the boundary layer, T_v changes little with height, supporting the use of surface parameters to approximate the $\overline{T_v}$. The spatial map of the derived Pt_{BL} is shown in Supplementary Figure 11.

In Eq. (37), the q_s is calculated from the 37-level specific humidity field (q) in the ERA5 reanalysis based on surface pressure (Ps). If $P_n \leq P_s \leq P_{n+1}$, where P_n = 1000, 975, 950, …, 2 hPa (for n = 1, 2, 3, …, 36), then

$$q_s = q(P = P_{n+1}), \quad (38)$$

If Ps > 1000 hPa, we set

$$q_s = q(P = 1000\ hPa), \quad (39)$$

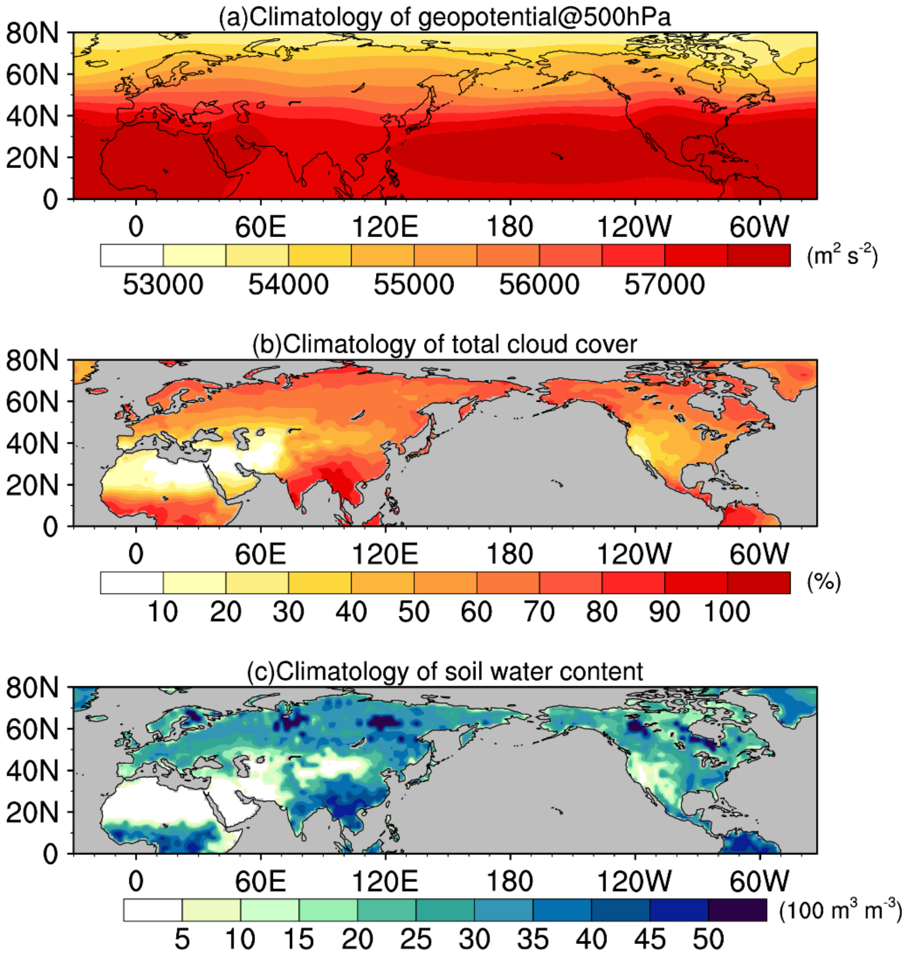

Supplementary Figure 1. Seasonal-mean atmospheric variables: (a) geopotential at 500 hPa (shading; $m^2 s^{-2}$), (b) total cloud cover (shading; %) and (c) soil water content (shading; 100 $m^3 m^{-3}$).

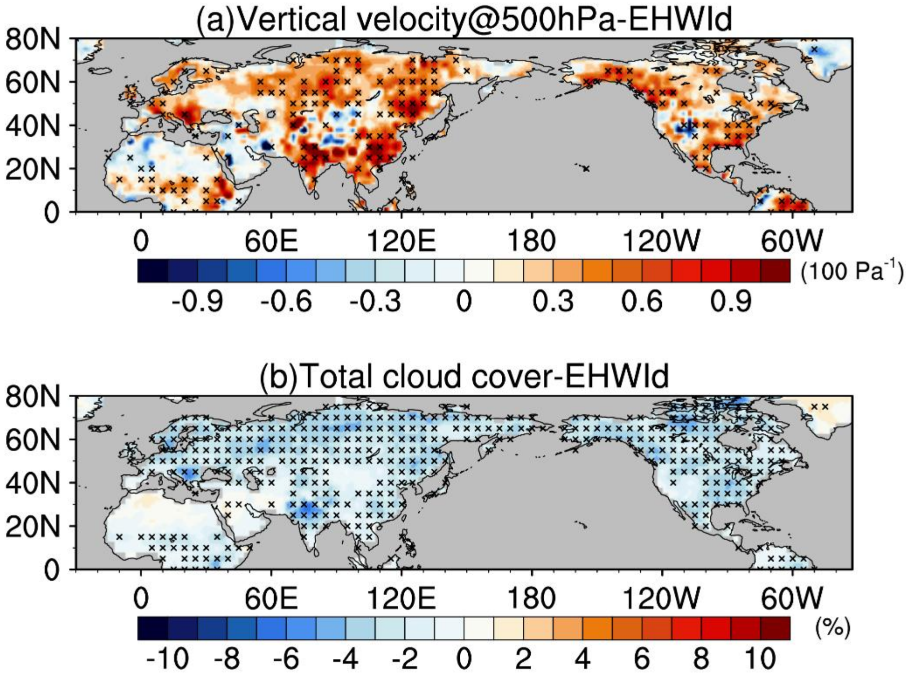

Supplementary Figure 2. Anomalies of vertical velocity at 500 hPa and total cloud cover (TCC) associated with extreme heatwaves. Anomalies of (a) vertical velocity at 500 hPa (shading; 100 Pa^{-1}) and (b) TCC (shading; %) obtained through pointwise regression onto the $EHWI_d$. Black crosses mark grid points where anomalies exceed the 95% confidence level (Student's *t*-test).

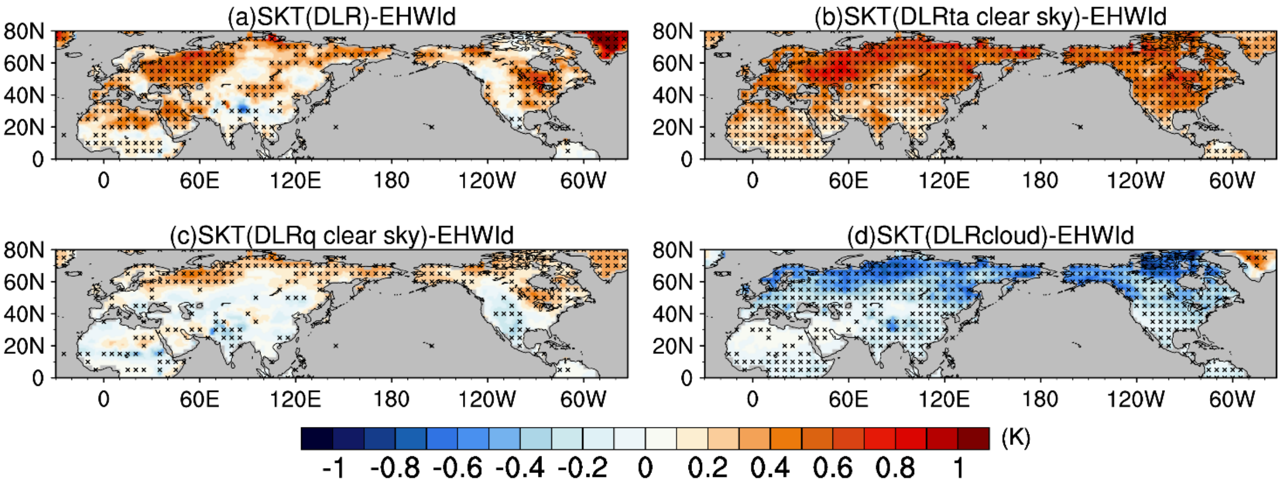

Supplementary Figure 3. Skin temperature (SKT) anomalies attributed to downwelling longwave radiation (DLR) and its contributing factors, diagnosed using a framework that combines the land surface energy budget, the Stefan–Boltzmann law, and an ERA5 based radiative kernel approach. Pointwise regressions onto the co-located $EHWI_d$ are shown for (a) DLR (SKT_{DLR}), (b) air temperature-related DLR changes (SKT_{DLRta}, clear-sky), (c) water vapor-related DLR changes (SKT_{DLRq}, clear-sky), and (d) cloud-related DLR changes (SKT_{DLRcld}; defined as the all-sky minus clear-sky SKT_{DLR}). Shading denotes SKT anomalies (K), and black crosses indicate grid points significant at the 95% confidence level (Student's *t*-test).

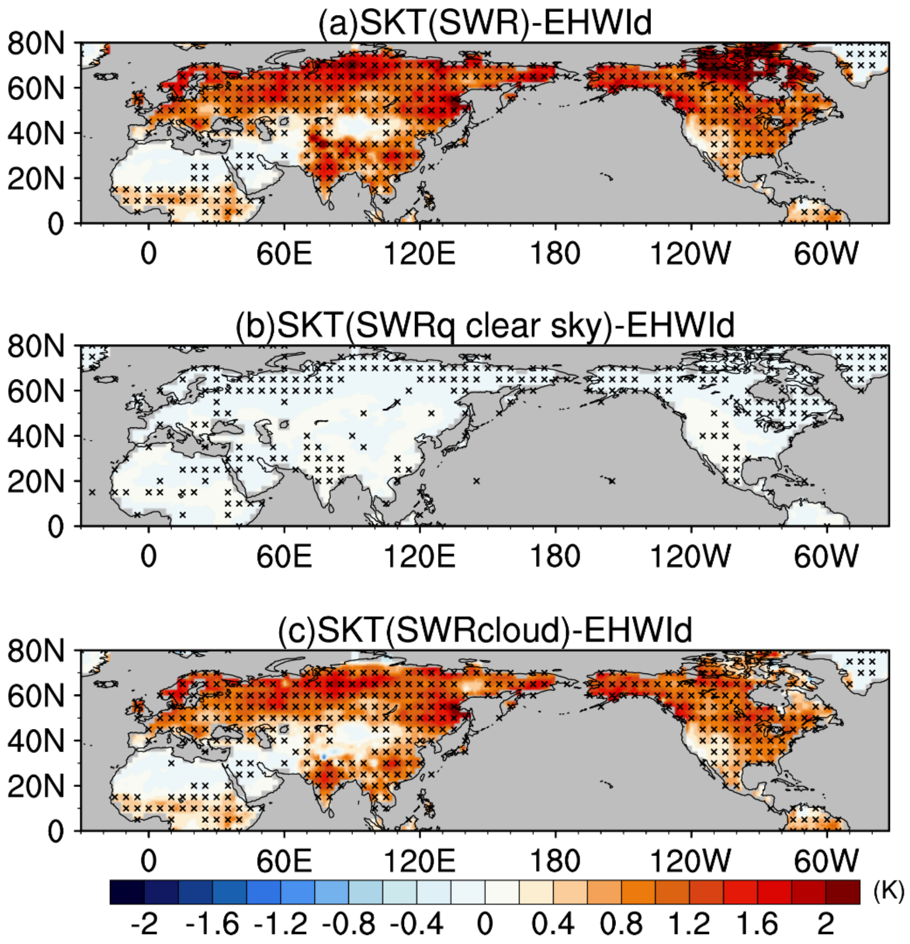

Supplementary Figure 4. Skin temperature (SKT) anomalies attributed to net surface shortwave radiation (SWR) and its contributing factors, diagnosed using a framework that combines the land surface energy budget, the Stefan–Boltzmann law, and an ERA5 based radiative kernel approach. Pointwise regressions onto the co-located $EHWI_d$ are shown for (a) SWR (SKT_{SWR}), (b) water vapor-related SWR changes (SKT_{SWRq}, clear-sky), and (c) cloud-related SWR changes (SKT_{SWRcld}; defined as the all-sky minus clear-sky SKT_{SWR}). Shading denotes SKT anomalies (K), and black crosses indicate grid points significant at the 95% confidence level (Student's *t*-test).

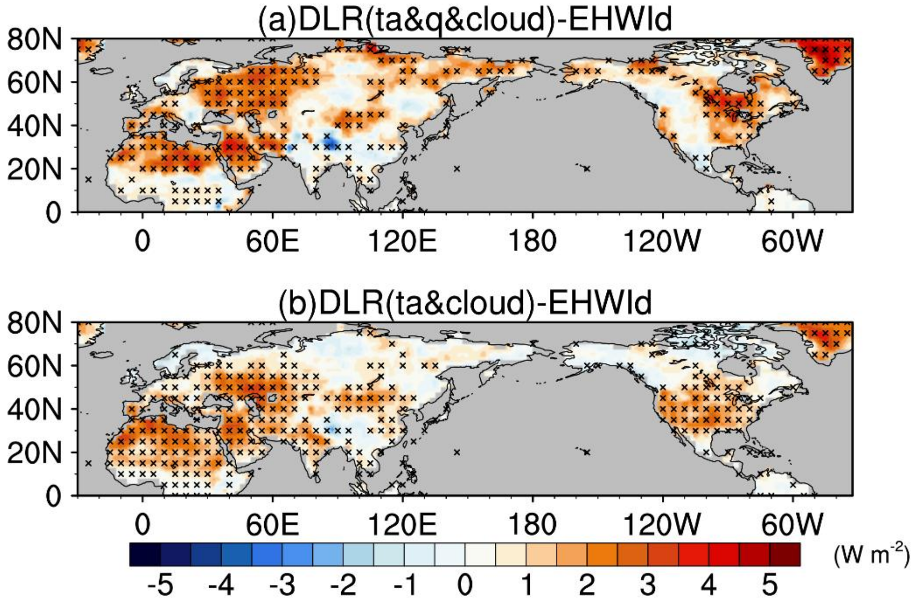

Supplementary Figure 5. The role of atmospheric water vapor in DLR anomalies associated with extreme heatwaves. Pointwise-regressed DLR anomalies onto the $EHWI_d$, comparing (a) the total DLR anomalies, obtained by combining the effects of air temperature, atmospheric water vapor, and clouds, with (b) anomalies derived from air temperature and cloud effects only. Shading denotes DLR values in W m^{-2}, and black crosses mark grid points significant at the 95% confidence level (Student's *t*-test).

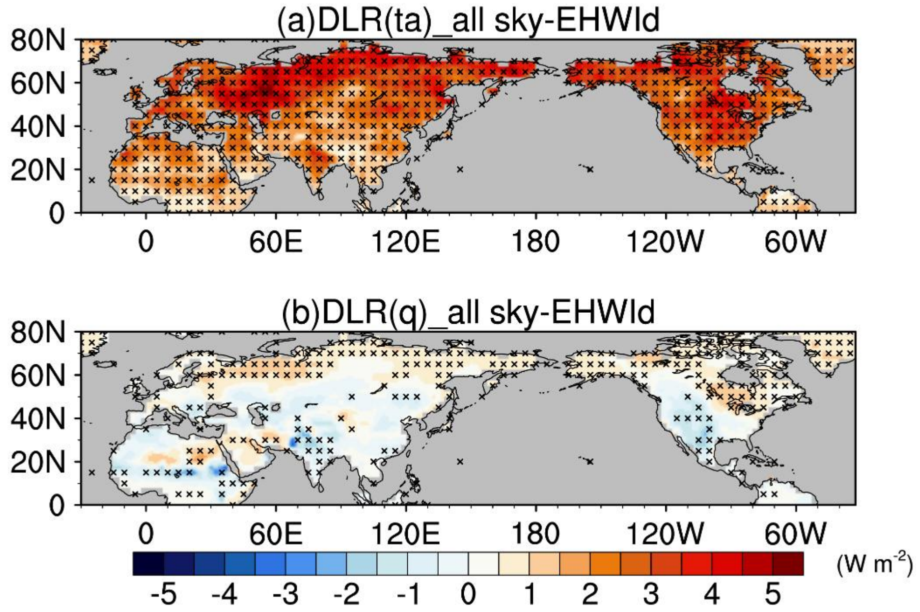

Supplementary Figure 6. The contributions of air temperature and atmospheric water vapor to DLR under all-sky conditions. Pointwise-regressed DLR anomalies onto the $EHWI_d$, showing contributions from (a) air temperature and (b) atmospheric water vapor. Shading denotes DLR values in W m^{-2}, and black crosses mark grid points significant at the 95% confidence level (Student's *t*-test).

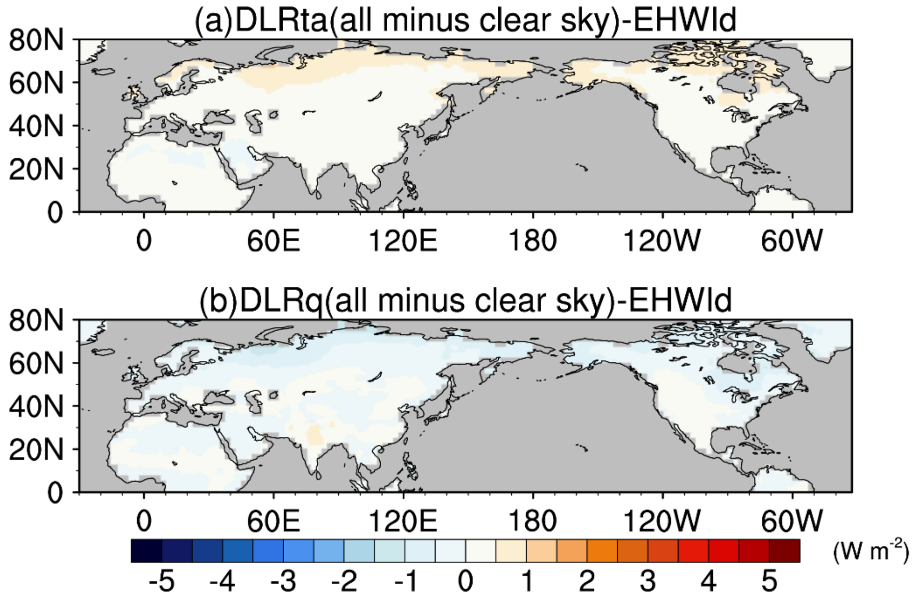

Supplementary Figure 7. Mean-state cloud modulation of DLR responses to air temperature and water vapor. Cloud-induced modulation of DLR responses attributed to (a) air temperature and (b) atmospheric water vapor, computed as the differences between all-sky and clear-sky kernel-derived DLR responses. Shading denotes DLR values in W m^{-2}.

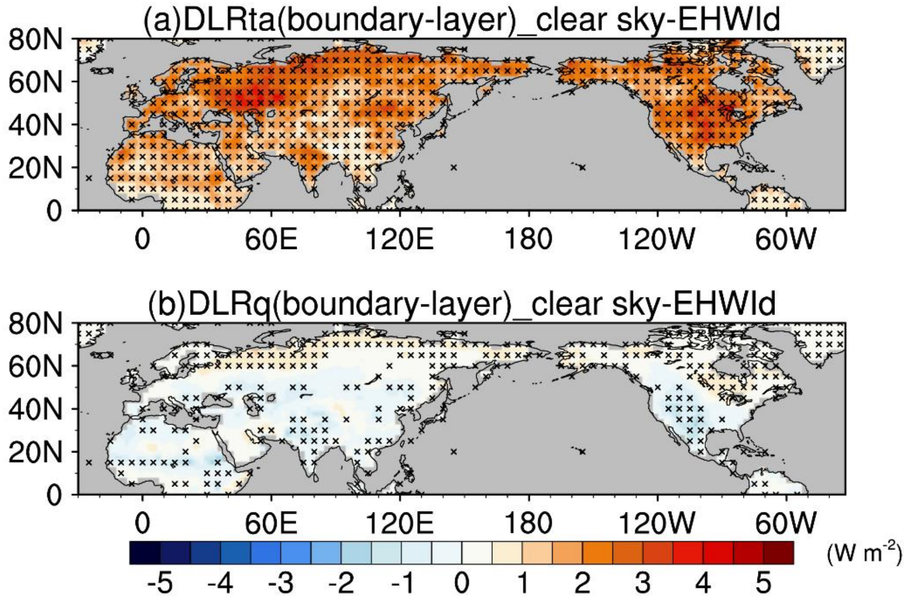

Supplementary Figure 8. The contributions of air temperature and atmospheric water vapor to DLR under clear-sky conditions, with the upper pressure bound of the mass integral set to the boundary-layer top pressure. Pointwise-regressed DLR anomalies onto the $EHWI_d$, showing contributions from (a) air temperature and (b) atmospheric water vapor. Shading denotes DLR values in W m^{-2}, and black crosses mark grid points significant at the 95% confidence level (Student's *t*-test).

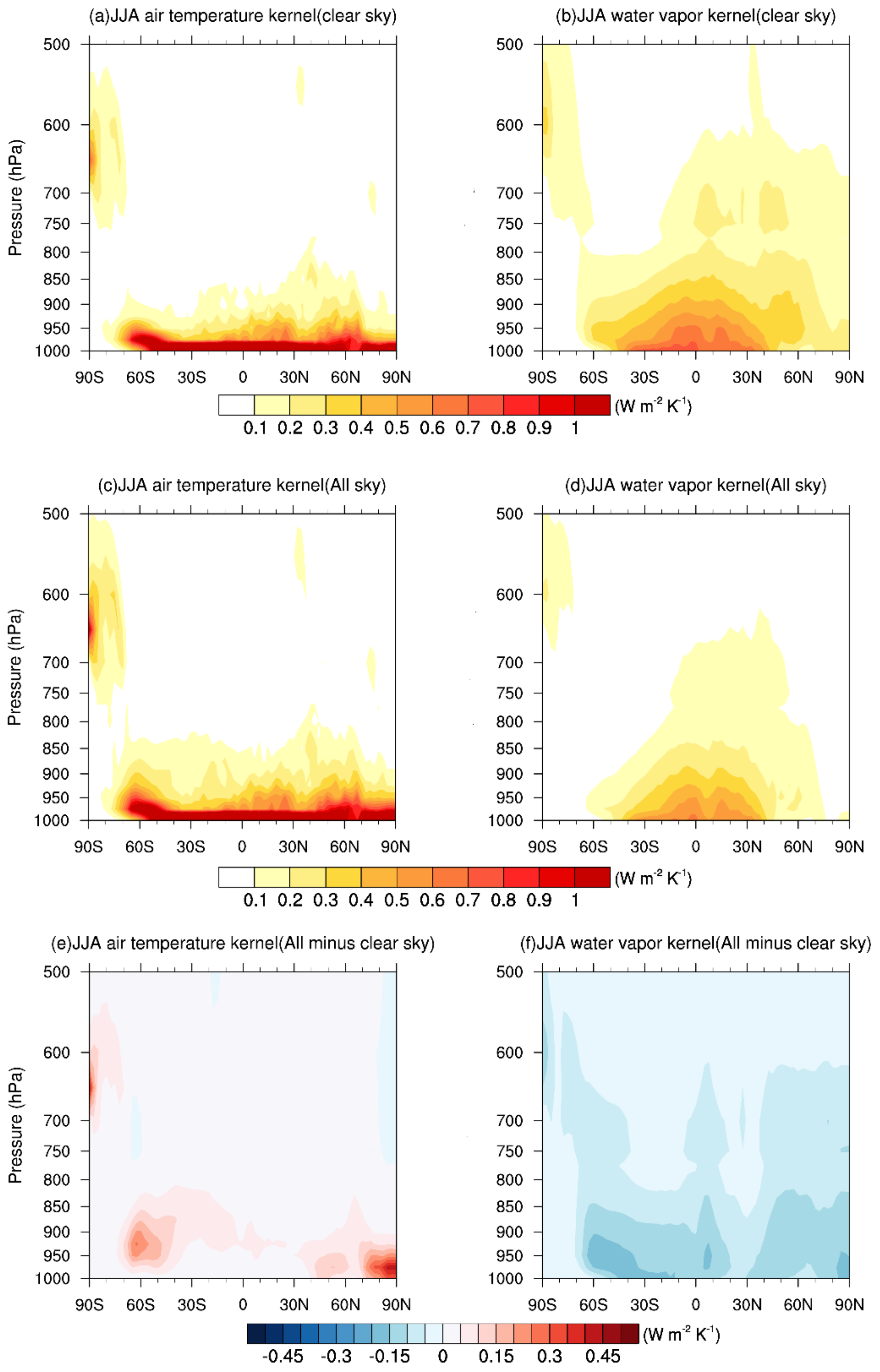

Supplementary Figure 9. Surface radiative kernels for DLR from ERA5 reanalysis. Vertical profiles of zonally averaged surface radiative kernels under (a, b) clear-sky and (c, d) all-sky conditions, with respect to (a, c) air temperature and (b, d) specific humidity, together with differences between all-sky and clear-sky kernels for (e) air temperature and (f) specific humidity. Shading indicates values (W m^{-2} K^{-1}) as a function of pressure level.

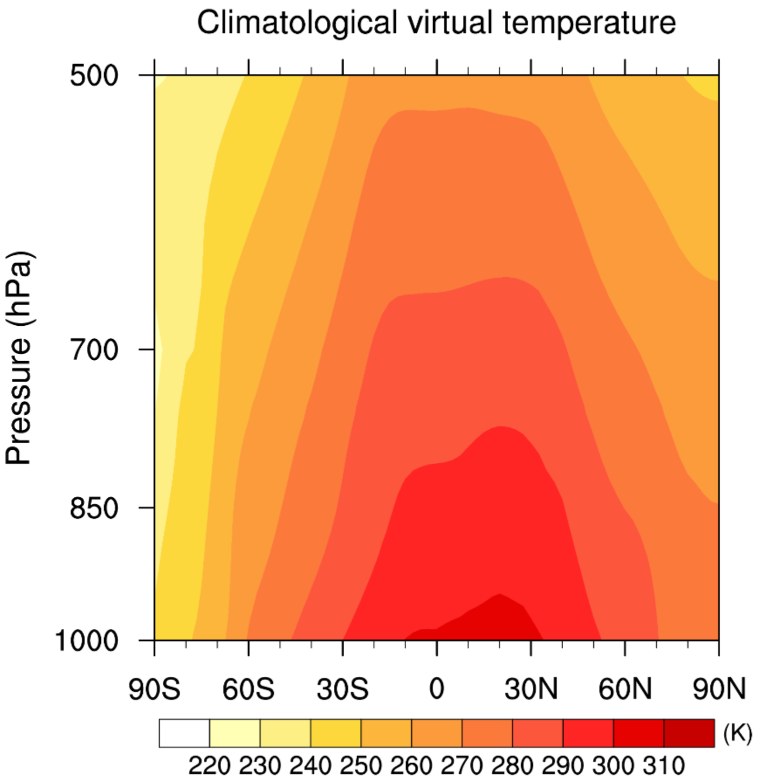

Supplementary Figure 10. Climatological virtual temperature. Vertical profiles of zonally averaged JJA-mean virtual temperature. Shading indicates virtual temperature values (K).

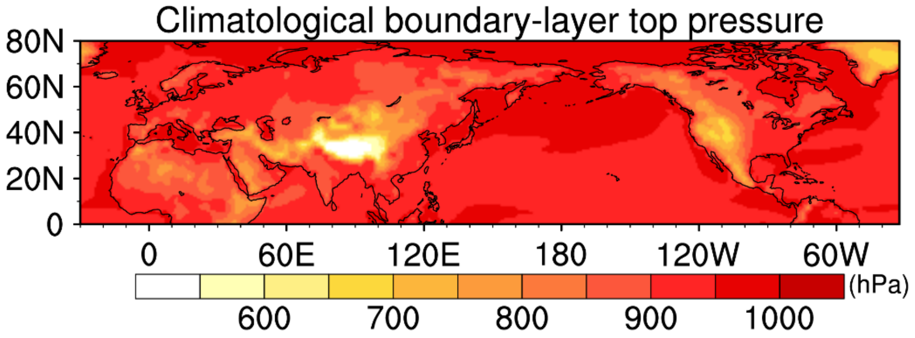

Supplementary Figure 11. Climatological boundary-layer top pressure (Pt_{BL}) during JJA. Shading denotes Pt_{BL} values (hPa).

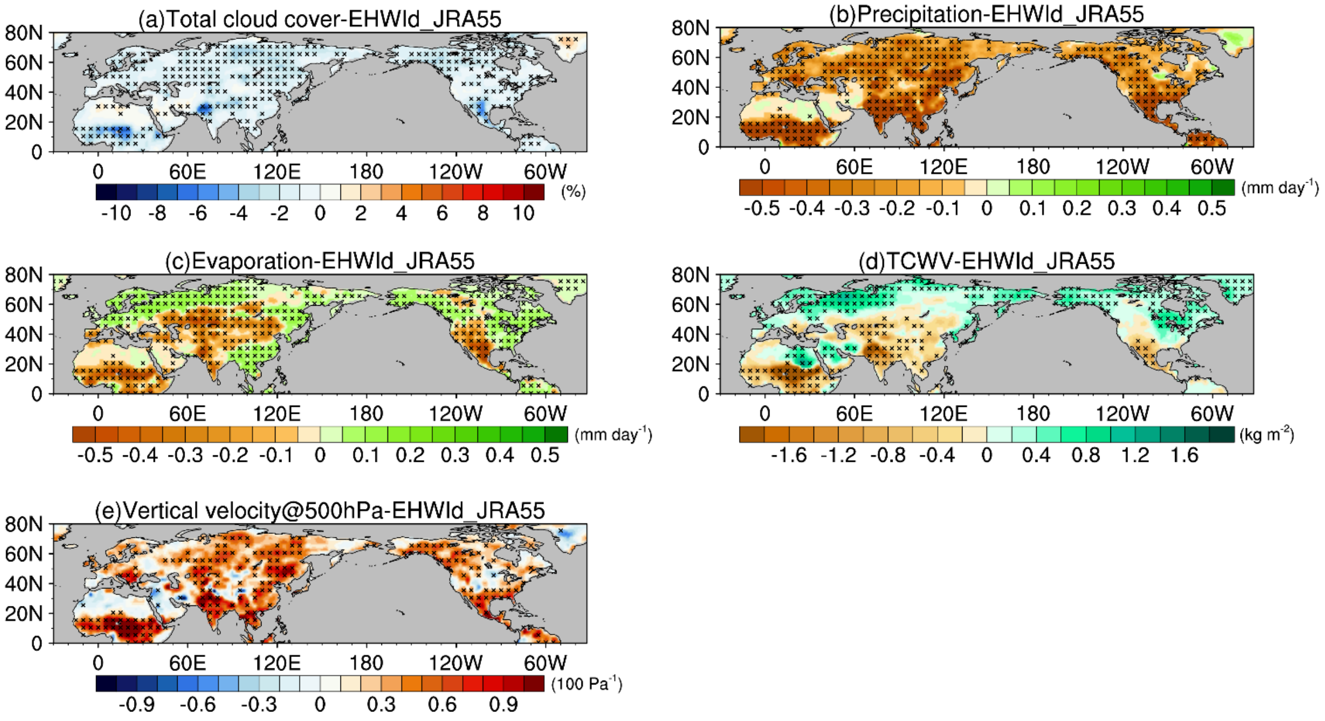

Supplementary Figure 12. Anomalies of atmospheric variables associated with extreme heatwaves obtained from JRA-55 reanalysis. Anomalies of (a) TCC (shading; %), (b) precipitation (shading; mm day^{-1}), (c) surface evaporation (shading; mm day^{-1}), (d) TCWV (shading; kg m^{-2}) and (e) vertical velocity at 500 hPa (shading; 100 Pa^{-1}) obtained through pointwise regression onto the EHWI$_d$. Black crosses mark grid points where anomalies exceed the 95% confidence level (Student's *t*-test).

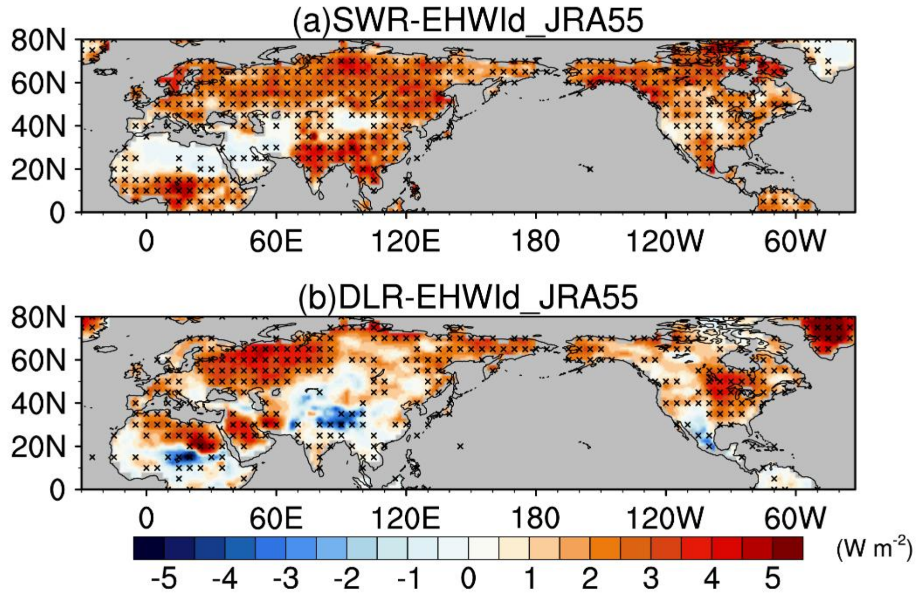

Supplementary Figure 13. Anomalies of surface radiative fluxes associated with extreme heatwaves obtained from JRA-55 reanalysis. Pointwise regressions onto the co-located $EHWI_d$ for (a) DLR and (b) SWR. All variables are shown as shading (W m^{-2}). Black crosses indicate values significant at the 95 % confidence level (Student's *t*-test).
